# The hyaluronan-related genes HAS2, HYAL1-4, PH20 and HYALP1 are associated with prognosis, cell viability and spheroid formation capacity in ovarian cancer

**DOI:** 10.1007/s00432-022-04127-6

**Published:** 2022-06-29

**Authors:** Jette Riecks, Arianna Parnigoni, Balázs Győrffy, Ludwig Kiesel, Alberto Passi, Davide Vigetti, Martin Götte

**Affiliations:** 1 Department of Gynecology and Obstetrics, Münster University Hospital, Albert-Schweitzer-Campus 1, 11, 48149 Münster, Germany; 2 Department of Medicine and Surgery, University of Insubria, Varese, Italy; 3 Department of Bioinformatics, Semmelweis University, Budapest, Hungary; 4 2nd Department of Pediatrics, Semmelweis University, Budapest, Hungary; 5 TTK Momentum Cancer Biomarker Research Group, Budapest, Hungary

**Keywords:** Ovarian cancer, Hyaluronidases, Hyaluronan synthases, HAS2, Gene expression, Survival analysis

## Abstract

**Purpose:**

Hyaluronan modulates tumour progression, including cell adhesion, cohesion, proliferation and invasion, and the cancer stem cell phenotype. In ovarian cancer, high levels of stromal hyaluronan are associated with poor prognosis. In this work, hyaluronan synthases (HAS1-3) and hyaluronidases (HYAL1-4, PH-20, HYALP1) were examined with regard to different levels of gene expression and its influence on ovarian cancer patients’ survival. The impact of a siRNA depletion of HAS2 was investigated in vitro.

**Methods:**

Using the Kaplan–Meier Plotter tool, we investigated the influence of hyaluronic synthases and hyaluronidases on the survival of a collective of 1435 ovarian cancer patients. Differences in gene expression between normal (*n* = 46) and cancerous (*n* = 744) ovarian tissue were examined using the TNMplot database. Following an evaluation of hyaluronan-related gene expression in the ATCC ovarian cancer panel, we studied SKOV3 and SW 626 ovarian cancer cells subjected to HAS2 siRNA or control siRNA treatment in terms of HAS1-3, HYAL2 and HYAL3 mRNA expression. We investigated the ability to form spheroids using the Hanging Drop method and the response to chemotherapy at different concentrations using the MTT Assay. By STRING analysis, interactions within the enzymes of the hyaluronic acid system and with binding partners were visualized.

**Results:**

HAS1, HYAL1 and HYAL4 mRNA expression is significantly upregulated, whereas HAS2, HYAL2 and HYAL3 mRNA expression is significantly downregulated in ovarian cancer tissue compared to controls. HAS2 improves cell viability, the capability to form tumour spheroids and has a negative prognostic value regarding overall survival. Lower HAS2 expression and high expression of HYAL2 and HYAL3 favours the survival of ovarian cancer patients. HAS2 knockdown cells and control cells showed a moderate response to combinatorial in vitro chemotherapy with taxol and cisplatin.

**Conclusion:**

In conclusion, our study shows that the hyaluronic acid system has a relevant influence on the survival of ovarian cancer patients and could therefore be considered as a possible prognostic factor.

## Introduction

Ovarian cancer is the second deadliest gynaecological tumour after breast cancer. 75% of carcinomas are detected at an advanced stage since the symptoms are very unspecific. In total, one of 72 women would be diagnosed with ovarian cancer. The relative 5-year survival rate is 43% (Wagner and Reuß 2019; Vitale et al. 2019a, b).

In 2020, the global incidence was 6.6 and the mortality was 4.2 referred to 100,000 people of all age groups (World Health Organization 2020). The common therapy is an operative resection or a systemic therapy that consists of a platinum-containing combination therapy (carboplatin/paclitaxel) or monotherapy. Patients who have a relapse in the first six months are platinum resistant. For these, a non-platinum-containing monotherapy (e.g. paclitaxel) is recommended. Patients without this resistance get platinum-containing combination therapy case of recurrence (Wagner and Reuß 2019; Vitale et al. 2019a, b).

The extracellular matrix (ECM) plays an important role in regulating cancer progression of different tumours. Indeed, many of them are surrounded by an ECM enriched with hyaluronan (HA), and ovarian cancer is no exception (Anttila et al. 2000; Hiltunen et al. 2002). High HA deposition correlates with a higher degree of both invasiveness and metastatic potential in ovarian tumours and reduces the efficacy of chemotherapeutics to induce cell death (Jojovic et al. 2002; Ricciardelli et al. 2013). HA is a glycosaminoglycan consisting of repeating disaccharide chains of *N*-acetyl glucosamine and d-glucuronic acid (Garantziotis and Savani 2019; Ween et al. 2011; Tavianatou et al. 2019). Through interaction with cell surface receptors (e.g. CD44 and RHAMM) and HA-binding proteins (e.g. TSG6), HA plays important roles in regulating diverse cell behaviours, including cell adhesion, proliferation, migration, and differentiation (Bourguignon et al. 2001; Lee and Spicer 2000; Vitale et al. 2019a, b). HA is synthesised at the plasma membrane by three isoenzymes called HA synthases (HAS1, HAS2 and HAS3), while its degradation is mediated by six known hyaluronidases called HYAL1-4, PH20 and HYALP1 (Itano et al. 1999; Csoka et al. 2001). The three HASs show differences in terms of tissue distribution and enzymatic properties; indeed, they produce HA molecules of diverse average sizes and show different synthesis rates. Nevertheless, they are similar in amino acid sequences and molecular structures (Tavianatou et al. 2019; Itano et al. 1999). Even though the precise role of each HAS in biological processes is still under investigation, it has been well demonstrated that HAS2 is the most important and catalytically active HA synthesizing enzyme, being highly expressed in several adult tissues and essential for the successful development of several organs (e.g. heart and limbs) (Camenisch et al. 2000; Matsumoto et al. 2009). Its expression is finely regulated from epigenetics to transcriptional and post-translational modifications, and its synthetic activity is normally related to the production of high-molecular-mass HA (Passi et al. 2019; Caon et al. 2021). On the other hand, HAS1 is the least active HAS, whereas HAS3 mostly synthesizes low-molecular-mass HA (Triggs-Raine 2015; Itano et al. 1999). HA size is also dependent on the activity of HYALs. HYAL1 and 2 are the two predominant isoforms cleaving HA: HYAL2 cleaves high-molecular-mass HA at the plasma membrane, which fragments are internalized into the cells and further degraded into smaller fragments via HYAL1 in the lysosomes (Csoka et al. 2001; Caon et al. 2021). Depending on its size, HA can exert different functions: high-molecular-mass HA promotes anti-inflammatory, anti-proliferative and anti-angiogenic effects, whereas low-molecular-mass HA are more prone to stimulate intracellular pathways promoting inflammation, cell proliferation and angiogenesis (Cuff et al. 2001; Tavianatou et al. 2019). Small HA fragments are mostly produced during pathological conditions, such as fibrosis, inflammation and cancer, and act as cellular alarm signals (Schmaus et al. 2014; Anders and Schaefer 2014).

Some previous studies have pointed to the importance of HASs and HYALs in ovarian cancer, which are reviewed below. Ilana Weiss et al. found the following by clinicopathologic data of 25 patients with primary serous ovarian cancer. Expression was measured by quantitative PCR (qPCR) and was compared between effusions, primary tumours and solid metastases. Metastases were from the omentum, uterus or lymph node. The results show that HAS1 was overexpressed in effusions, HAS2 was overexpressed in solid metastases and primary tumours, and HAS3 was overexpressed in primary carcinomas and effusions. HYAL1 could not be detected at all. HYAL2 was present in two variants. The expression of HYAL3 was high in primary carcinomas and effusions. In addition, a change in expression due to chemotherapy treatment was detected in this project for primary carcinomas. Twenty two tumours were obtained before treatment with chemotherapy and 3 after neoadjuvant chemotherapy. HAS1 was less expressed in effusions after treatment with platin and after treatment with paclitaxel. HYAL3 was more expressed after treatment with paclitaxel. High HYAL2-var1 expression correlated with longer overall survival and high HAS1 expression correlated with lower overall survival (Weiss et al. 2012). Another group demonstrated that HAS1 appears to have an impact on angiogenesis in ovarian cancer, which also negatively correlates with overall survival. Furthermore, they found that the expression of HAS1-3 has no influence on the response to chemotherapy (Yabushita et al. 2004). Finally, it was found that even in tissues that do not normally contain HA, the level of HA increases as the malignancy of the tumour increases. This also applies to the tumour stroma, as in ovarian carcinoma. Overall, this HA accumulation correlates with a poor prognosis for the patient (Tammi et al. 2008). While these studies indicate a potentially important role for HA synthesis and degradation in ovarian cancer, no comprehensive analysis of the prognostic impact of HASs and HYALs in a large collection of ovarian cancer patients has been performed. Moreover, the functional impact of the major hyaluronan synthase HAS2 in ovarian cancer cells is so far unclear.

This work collects, for the first time, a comprehensive analysis of the survival of patients and the aggressiveness of ovarian cancer, collecting data on all the main genes involved in the metabolism and signalling of HA (i.e., HASs, HYALs, HA-receptors and HA-interacting molecules). In particular, in this project, potential differences in gene expression between tumour and normal tissues, and the influence of gene expression of HA enzymes on overall survival and progression-free survival of ovarian cancer patients was analyzed by using the Kaplan–Meier-Plotter online database comprising gene expression and survival data of 1435 ovarian cancer patients (Győrffy et al. 2012). At the molecular level, we evaluated basal expression levels of the HA axis in a panel of ovarian cancer cell lines and compared control ovarian cancer cells and HAS2 knockdown cells regarding gene expression of HAS1-3 and HYAL2-3, CD44, RHAMM and versican by qPCR. Moreover, we analyzed the ability to form spheroids by using the Hanging Drop method. We furthermore investigated the cell viability of HAS2 knockdown cells compared to control cells under treatment with different chemotherapy concentrations by MTT assay, and evaluated a potential impact on cell motility.

## Materials and methods

### The human protein atlas

The human protein atlas V21.0 was used to analyse the protein expression, of the genes that were evaluated by the Kaplan Meier Plotter, in ovary tissue by immunohistochemistry (HPA, https://www.proteinatlas.org, accessed on 24.02.2022) (Uhlén et al. 2015). The intensity of the immunohistochemical staining for tumour tissue is divided into “high”, “medium”, “low” and “not detected”. The antibodies HPA067602 for HAS1, CAB033850 for HAS3, HPA002112 for HYAL1, HPA036436 for HYAL2, HPA049402 for HYAL3, HPA029453 for HYAL4 and HPA017984 for PH20 were used.

### TNMplot analysis

The TNMplot is an online available database that shows gene expression ranges for different tissues (https://tnmplot.com/analysis/, accessed on 24.02.2022), (Bartha and Győrffy 2021). We used this analysis for the gene products that were analysed by the Kaplan–Meier Plotter and evaluated their mRNA expression levels for control ovary tissue (*n* = 46) and ovarian cancer samples (*n* = 744) analysing gene chip data. For this platform, the setting “use non-paired tumour and normal tissues” was chosen.

### Kaplan–Meier Plotter analysis

Kaplan–Meier Plotter (https://kmplot.com/analysis/, accessed on 11.04.2021) is a publicly accessible database that integrates gene expression data and survival information of 1435 ovarian cancer patients downloaded from the public repository Gene Expression Omnibus (GEO) (Győrffy et al. 2012). The tool allows for analysing the overall survival (OS) and progression-free survival (PFS) of ovarian cancer patients using different stratifications. Related to the enzymes of the HA system of ovarian cancer patients, there is data of 1435 patients for OS and 655 for PFS. The patient data is divided into two subgroups split by the median of target gene expression: patients with high expression and patients with low expression of the gene. In this project, patient data were evaluated in different subgroups referred to by histology, stage, grade, and various chemotherapy treatments. The analysis of ovarian cancer patients was carried out for the enzymes HAS1, HAS2, HAS3, HYAL1, HYAL2, HYAL3, HYAL4, PH20 and HYALP1 of the HA system. The Affymetrix ID for the genes are 207316_at for HAS1, 206432_at for HAS2, 223541_at for HAS3, 210619_s_at for HYAL1, 206855_s_at for HYAL2, 211728_s_at for HYAL3, 220249_at for HYAL4, 210536_s_at for PH20 and 1564777_at for HYALP1.

### Cell culture

The human ovarian cancer cell lines SKOV3, SW 626, PA-1 and Caov-3 (Ovarian Cancer Panel, TCP-1021™) were purchased from ATCC/LGC Promochem (Wesel, Germany). SKOV3 cells were cultured in McCoy´s 5A medium (Sigma-Aldrich®, prod. no. M9309, MDL no. MFCD00217560 Saint Louis, USA) containing 10% foetal calf serum (FCS) (Pan biotech™, cat. no. P40-37,500, Germany) and 1% Penicillin/Streptomycin (Sigma-Aldrich®, cat. no. P4333, Saint Louis, USA) and were maintained in a humidified atmosphere with 7.5% CO_2_ at 37 °C. SW 626 and Caov-3 cells were cultured in Dulbecco´s modified Eagle´s Medium (Sigma-Aldrich®, prod. no. D4947, Saint Louis, USA) containing 10% foetal calf serum (FCS) (Pan biotech™, cat. no. P40-37500, Germany). The cells were maintained at 37 °C and 100% air. PA-1 cells were cultured in Eagle´s Minimum Essential Medium (Pan biotech™, cat. no. P40-09500, Germany) containing 10% foetal calf serum (FCS) (Pan biotech™, cat. no. P40-37500, Germany). The cells were maintained at 37 °C in a humidified atmosphere with 5% CO_2_.

### siRNA transfection

3.5 × 10^5^ cells per well were cultured for 24 h in a complete medium, containing 10% FCS and 1% Penicillin/Streptomycin. For transfection, the cells were 60–70% confluent. First, the medium was replaced by 840 µl Opti-MEM®/well (Gibco®, cat. no. 31985–070, Thermo-scientific, Germany). Cells in each well were transfected with 80 µl 20 nM negative control siRNA/Opti-MEM® (Ambion®, cat. no. 4390844, Cambridgeshire, UK) or HAS2 siRNA/Opti-MEM® (Ambion®, cat. no. AM16708, ID 117,327, Cambridgeshire, UK) and 80 µl 2,5% Lipofectamin/Opti-MEM® reagent (Lipofectamine™ RNAiMAX Transfection Reagent, cat. no. 13778–075, Thermo-scientific, Germany). Cells got in the incubator at 37 °C and 7.5% CO_2_. After 24 h of incubation, the transfection mixture was changed to 2 ml/well complete medium containing 10% FCS and 1% Penicillin/Streptomycin.

### Quantitative real-time PCR

Total RNA was isolated from cells using the InnuPREP RNA mini kit (Analytikjena, cat. no. 845-KS-2040250, Jena, Germany). It was transcripted into cDNA carried out with the High-Capacity cDNA Reverse Transcription Kit (Applied Biosystems, cat. no. 4368814, Foster City, CA, USA) following the supplier´s protocols. qPCR was performed in a 7300 real-time PCR detection system (Applied Biosystems) with RT2 SYBR Green qPCR Primer Assay (Qiagen, cat. no. 330500, Hilden, Germany) and Takyon™ ROX probe qPCR Kit (Eurogentec GmbH, cat. no. UF-RPMT-B0100, Cologne, Germany). HAS2 knockdown was confirmed using the TaqMan probe HS00193435 m1 (Applied Biosystems). Results were evaluated using the 2^−∆∆Ct^ method. β-actin samples were used as internal controls. The fold change shows the expression of the investigated enzymes in HAS2 knockdown cells compared to the control samples. Primer sequences are shown in Supplementary Table S1. The results were formed out of 7 experiments with double or triple replicates in each experiment.

### Particle exclusion assay

To evaluate the pericellular coat of HA, a particle exclusion assay was performed (Vigetti et al. 2009). Briefly, 3.5 × 10^5^ cells were seeded in a 6-well plate and transfected with 20 nM negative control siRNA or HAS2 siRNA. After 24 h, 2 × 10^7^ fixed human red blood cells were added to each well. After an incubation time of 30 min at 37 °C, cells were examined by phase contrast microscopy, and 10 pictures per well were taken. As a control, cells were treated with 2 U/ml of Hyaluronidase from *Streptomyces hyalurolyticus* (Sigma-Aldrich®, cat. No. H1136). The analysis of the images and the relative quantification were done using the image analysis software ImageJ (NIH, Bethesda, MD, USA).

### Hanging drop assay

The hanging drop method was used to measure cell cohesion and the ability to form spheroids of HAS2 knockdown cells compared to control cells. A 250 µl solution consisting of medium and 2.5 × 10^5^ cells was prepared. For each experiment, 12 drops with a volume of 20 µl were placed on the inside of the lid of a Petri dish. 10 mL of sterile PBS were placed on the bottom of the Petri dish. Due to this, the drops did not dry out. The Petri dish was placed in the incubator at 37 °C and 7.5% CO_2_. Pictures of the drops were taken with a ZEISS® Axiophot (Zeiss, Jena, Germany) bright-field microscope (magnification 5x) on day 4 and day 7 to visualize the form and size of the spheroids. This was done separately for control cells and HAS2 knockdown cells under the same conditions in four experiments with 12 drops each. Then, the area and perimeter of the spheroids per drop were measured by using NIH ImageJ software (Rasband and Image 1997–2018). Results were independently evaluated by two observers (Jette Riecks, Birgit Pers) with similar results.

### MTT assay

In a 96-well plate, 2000 cells were added to each well with 200 µl DMEM Medium (Gibco®, cat. no. 21063–029, ThermoFisher Scientific, Germany) containing 10% FCS. After 24 h of incubation at 37 °C and 7.5% CO_2_ a defined amount of chemotherapy was given in every well. The various chemotherapeutic agents were added to the well rows in decreasing concentrations. The last well of each dilution series had a concentration of 0.00 nM chemotherapy and served as a control. For taxol, the first well had a concentration of 1000 nM. This was reduced to 0.941 nM taxol via an 11-part 1:2 dilution series. A dilution series was also applied for cisplatin. The starting concentration was here 6.6656 nM and the final concentration was 0.007 nM. For the combination of taxol and cisplatin, the starting concentration was 42.9 nM taxol and 4.761 nM cisplatin. The final stage of the dilution series was 0.0419 nM taxol and 0.0046 nM cisplatin. After 72 h of incubation at 37 °C and 7.5% CO_2_ the medium was removed and cells were incubated for 4 h with 20 µl/well of 3-(4,5-dimethylthiazol-2-yl)-2,5-diphenyl-tetrazolium bromide (MTT) at 5 mg/ml. After that, the reaction was stopped by adding 100 µl Stopping solution/well. The stopping solution consisted of *N*,*N*-dimethylformatid (Sigma-Aldrich®, cat. no. 605365). The absorbance was measured in a VersaMax® Microplate Reader (Molecular Devices, Sunnyvale, CA, USA) at a wavelength of 595 nm. For data visualization, absorbances of all measured values were expressed as %, with 100% corresponding to the measured absorbance of the control cells at 0.00 nM chemotherapy. The results represent the values of 3 experiments performed in duplicates.

### Wound healing assay

The wound healing assay was used to measure the cell ability to migrate after HAS2 knockdown. Forty eight h after transfection, the cells were washed one time with 1X PBS, and then a scratched area was created using a sterile 200 μL pipette tip on 90% confluence, followed by incubation in serum-free complete medium for 24 h. Cells migrated into the wound surface were determined under the microscope at time intervals of 1–6 h. The ratio of cell migration was calculated as the percentage of closed wound compared with the area of the initial scratched area.

### STRING analysis

STRING v11.5 (https://string-db.org, accessed on 02.09.2021) is an online bioinformatic tool to analyze in silico protein interaction networks (Csóka et al. 1999). We carried out this analysis with the enzymes evaluated by the Kaplan–Meier-Plotter analysis. STRING uses classification systems like Gene Ontology (GO) and Kyoto Encyclopedia of Genes and Genomes (KEGG). The interactions were predicted with a medium confidence threshold of 0.400. All predictive methods were allowed (Szklarczyk et al. 2019).

### Statistical analysis

The statistical analysis of the Kaplan–Meier-Plotter was performed using the R statistical environment with the statistical package ‘survival’. The Kaplan–Meier-Plotter showed the influence of different expression levels of enzymes on the chance of survival by using Kaplan–Meier survival curves, the Hazard Ratio and the corresponding *p*-value (Győrffy et al. 2012), (Grillo et al. 2021). For qPCR, hanging drop and MTT Assay statistical analysis was performed using Microsoft Excel. Due to the two-tailed *t*-test, the *p* values were determined. *p* ≤ 0.05 is shown by *, *p* ≤ 0.001 by ** and *p* ≤ 0.0001 by ***.

## Results

### Evaluation of the hyaluronan biosynthetic and degradative axis using human protein atlas data

The human protein atlas V21.0 (Uhlén et al. 2015) was used to initially characterize the protein expression of the enzymes studied in this project comparing normal ovarian tissue with the expression in tumour tissue (Supplementary Figure S1). HAS1 was lowly expressed in normal tissue in ovarian stromal cells and was undetectable in follicular cells. In comparison, HAS1 expression in tumour cells was low to medium measured in 5 of 12 samples. HAS1 was not detected in the remaining samples. HAS2 could not be evaluated with the human protein atlas because protein data based on antibody staining were not available. HAS3 expression could not be detected in normal tissue in ovarian stromal cells and only to a low level in follicular cells. Tumour tissue showed a similar tendency. Low HAS3 expression was measured in one sample, while it was not detected in any of the other samples. HYAL1 and HYAL2 expression could not be detected in the ovarian stromal cells or follicular cells of normal tissue. In the tumour tissue, 3 of 12 and 11 samples, respectively, showed low to medium HYAL1 and HYAL2 expression, being undetectable in the remaining samples. For HYAL3 low-intensity expression was detected in follicular cells in normal tissue. No expression was shown for ovarian stromal cells. 3 of 11 samples showed low to medium expression in ovarian tumour tissue. HYAL4 could be detected at low intensity in ovarian stromal cells and not in follicular cells. In tumour tissue, all samples showed HYAL4 expression, with low expression in 8 samples and medium expression in 3 probes. PH20 expression could not be detected in 8 normal or tumour tissue using the antibodies and staining conditions utilized in the human protein atlas V21.0 project. In summary, normal tissue showed no or only a low expression of the investigated enzymes, whereas in tumour tissue a slightly increased expression of some of the enzymes was detectable (see Supplemental Figure S1 for details). High protein expression was not measured in any of the tissue samples for all enzymes examined.

### The gene expression levels of HAS1, HAS2 and HYAL1-4 are significantly altered in ovarian tumour tissue compared to control tissue

**Fig. 1:**
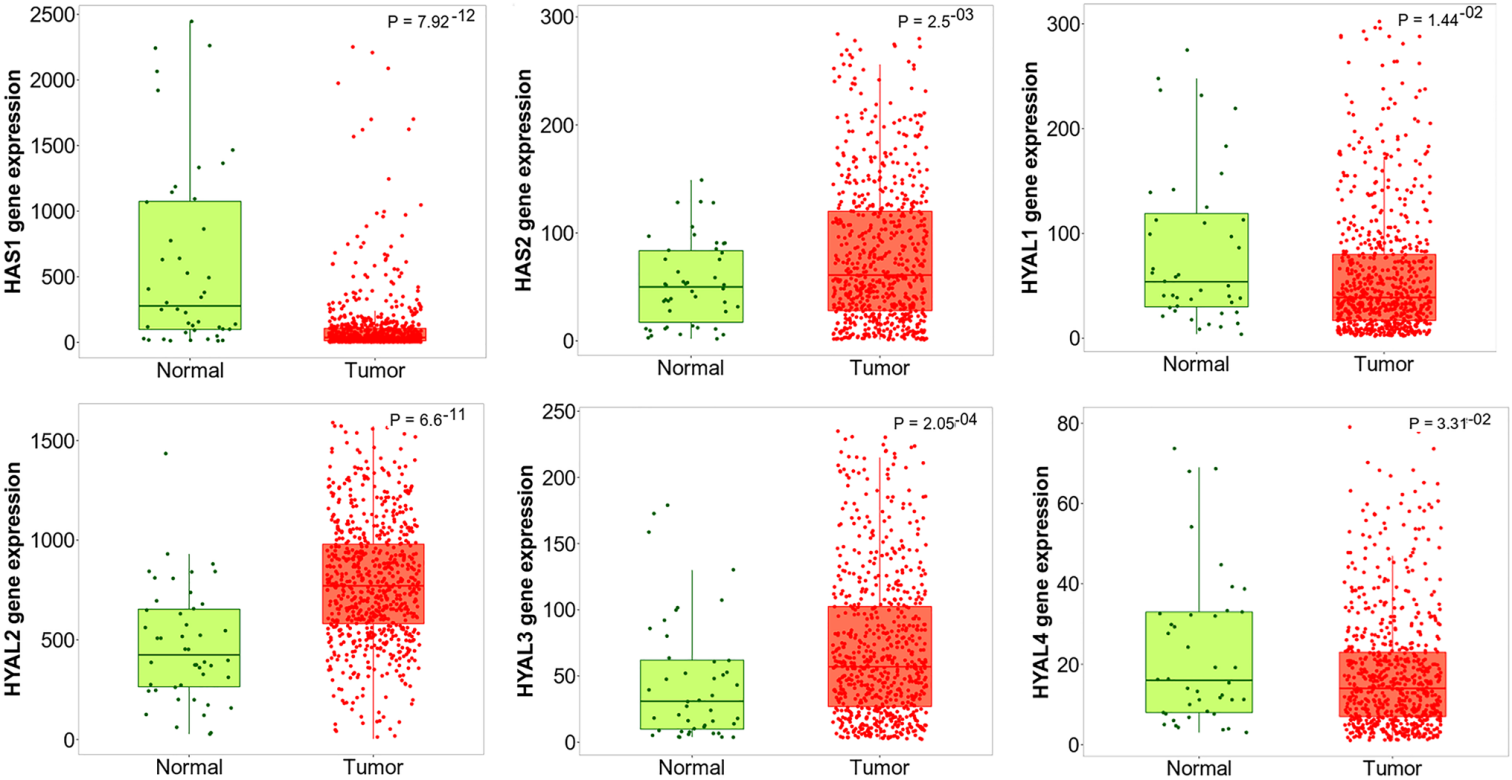
The gene expression levels of HAS1, HAS2 and HYAL1-HYAL4 of normal ovarian tissue (*n* = 46) compared with tumour tissue of the ovary (*n* = 744). While HAS1, HYAL1 and HYAL4 expression levels were significantly lower in tumour tissue compared to control ovary, the expression of HAS2, HYAL2, and HYAL3 were significantly upregulated in tumours compared to controls. The data are derived from the TNMplot database (Bartha and Györffy 2021) (https://tnmplot.com/analysis/, accessed on 24 Feb 2022)

We next aimed at investigating a potentially altered expression of the HA biosynthetic and degradative axis in a larger collective evaluating mRNA expression as a more robust means of quantification. The TNMplot database (Bartha and Győrffy 2021) was used to compare the gene expression of the enzymes studied in this project in normal tissue (*n* = 46) and tumour tissue (*n* = 744) of the ovary. The database did not include data for HAS3, PH20 and HYALP1, so these genes could not be examined. Significant changes in gene expression were seen for all genes examined, as shown in Fig. 1. HAS1 showed reduced gene expression in ovarian cancer tissue with a fold change of 0.11. The expression of HYAL1 (fold change = 0.69) and HYAL4 (fold change = 0.86) also was slightly reduced. In contrast, the expression of HAS2 (fold change = 1.42), HYAL2 (fold change = 1.84) and HYAL3 (fold change = 1.94) was significantly increased in the tumour tissue (Fig. 1).

### HAS2, HYAL2 and HYAL3 have a differential impact on the survival of ovarian cancer patients

**Fig. 2:**
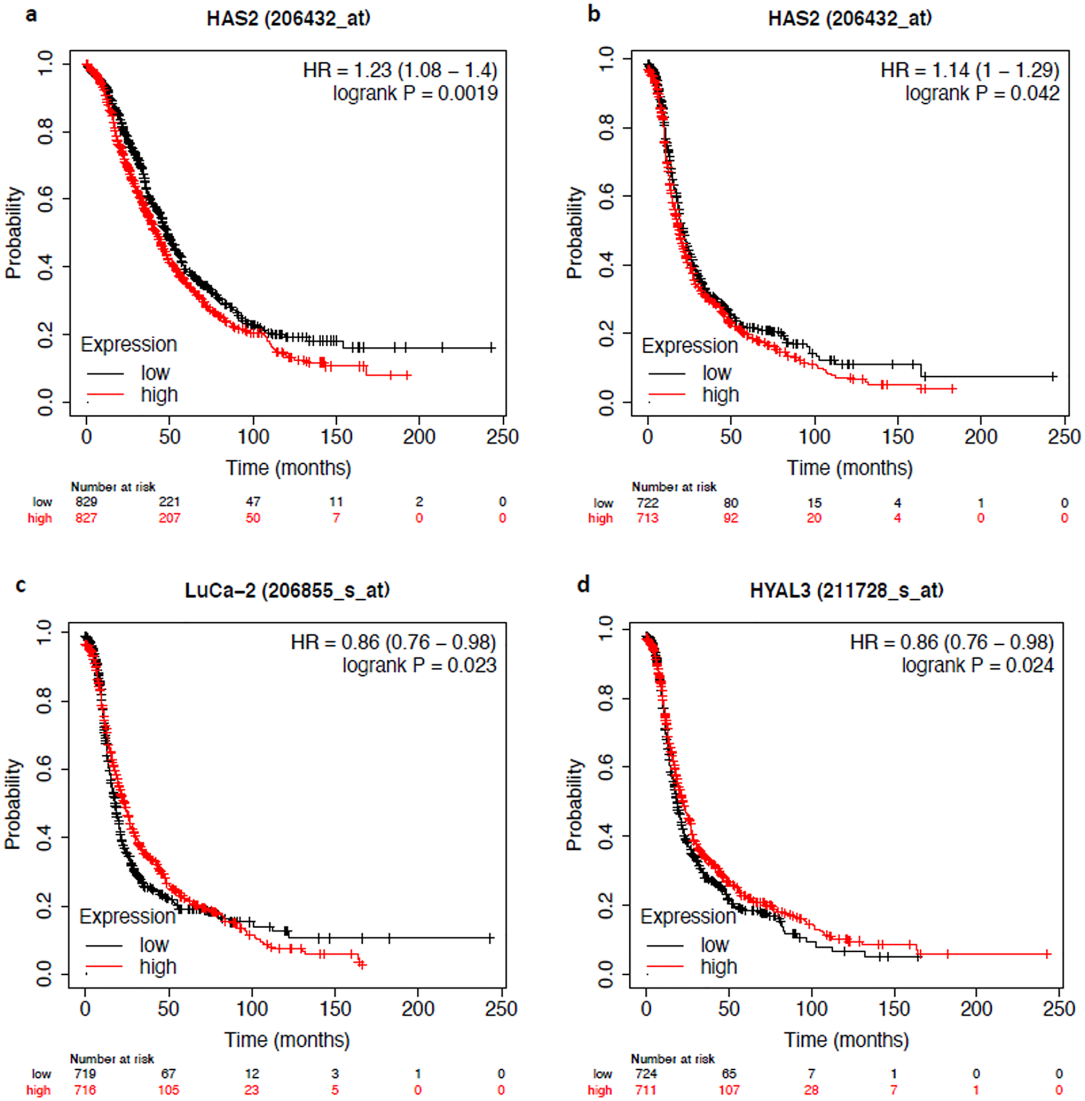
Prognostic value of HAS2, HYAL2 and HYAL3 for the survival of patients with ovarian cancer. The analysis was done by the Kaplan–Meier-Plotter. For each enzyme the Kaplan–Meier-curve, the hazard ratio (95% confidence interval) and the *p*-value were given. **a** OS in correlation with HAS2 expression (*n* = 1656), **b** PFS in correlation with HAS2 expression (*n* = 1435), **c** PFS in correlation with HYAL2 (LuCA-2) (*n* = 1435), **d** PFS in correlation with HYAL3 (*n* = 1435)

In this project, the Kaplan–Meier-Plotter database was used to present the influence of various enzymes of the HA system on the OS and PFS of patients. The number of specific patient cases per classification is shown in Table 1. The original patient collective was described in reference (Győrffy et al. 2012). HAS2 had a significant negative impact on survival in terms of both, OS and PFS (Table 1, Fig. 2a, b). The HR (hazard ratio) of OS was 1.23 (*p*-value = 0.0019) and the one of PFS was 1.14 (*p*-value = 0.042). A positive correlation was given to the high expression of HYAL2 and HYAL3 referred to PFS. The HR was 0.86 for both with a p-value of 0.023 for HYAL2 (Table 1, Fig. 2c) and 0.024 for HYAL3 (Table 1, Fig. 2d). Furthermore, subgroup analysis was done to find out whether enzymes had a particular stronger influence on certain patient groups. A distinction was made between histology, staging, grading and different chemotherapy approaches. Histology was subdivided into serous and endometrioid ovarian cancer, staging into stage I + II compared to III + IV and grading into grade I + II compared to III. The chemotherapy approaches were taxol compared to cisplatin and the combination of taxol and cisplatin. The results of these subgroup analyses are shown in Table 2 (HAS1-3), Table 3 (HYAL1-3) and Table 4 (HYAL4, PH20) and in Table S3 (HYALP1) in the supplement information.

**Table 1:** Correlation between the expression of HAS1-HAS3, HYAL1-HYAL4, PH20 and HYALP1 and the overall survival (OS) or progression free survival (PFS) of ovarian cancer patients

Genes	OS			PFS		
	Case *n*	HR	*p*-value	Case *N*	HR	*p*-value
HAS 1	1656	1.05	0.45	1435	1.1	0.13
HAS 2	1656	1.23	**0.0019**	1435	1.14	**0.042**
HAS 3	655	1	0.99	614	0.99	0.94
HYAL1	1656	1.05	0.49	1435	1.01	0.84
HYAL2	1656	0.9	0.099	1435	0.86	**0.023**
HYAL3	1656	0.88	0.046	1435	0.86	**0.024**
HYAL4	1656	0.95	0.4	1435	0.94	0.36
PH20	1656	1.02	0.79	1435	0.96	0.48
HYALP1	655	0.88	0.22	614	0.9	0.28

Data were analyzed by the Kaplan–Meier-Plotter. Number of cases, HR ad *p*-value are given. Statistically significant values are marked in bold typing

**Table 2:** Correlation between the expression of HAS1-HAS3 and the OS or PFS of ovarian cancer patients

Genes	OS			PFS		
	Cases	HR	*p*-value	Cases	HR	*p*-value
HAS 1						
Histology						
Serous	1207	1.02	0.79	1104	1.3	**0.00033**
Endometrioid	37	0.61	0.58	51	0.97	0.96
Staging						
I + II	135	0.71	0.38	163	1	0.99
III + IV	1220	1	0.96	1081	1.24	**0.0033**
Grading						
I + II	380	1.12	0.43	293	1.28	0.081
III	1015	1.05	0.55	837	1.29	**0.0029**
Chemotherapy						
Taxol + platin	776	1.07	0.49	698	1.25	**0.011**
HAS 2						
Histology						
Serous	1207	1.26	**0.0027**	1104	1.31	**0.00021**
Endometrioid	37	0	**0.013**	51	0.54	0.21
Staging						
I + II	135	0.96	0.91	163	1.47	0.19
III + IV	1220	1.18	**0.03**	1081	1.2	**0.012**
Grading						
I + II	380	1.44	**0.013**	293	1.28	0.081
III	1015	1.16	0.071	837	1.29	**0.0027**
Chemotherapy						
Taxol + platin	776	1.21	**0.045**	698	1.23	**0.019**
HAS 3						
Histology						
Serous	523	0.95	0.66	483	1	0.96
Endometrioid	30	0.19	0.11	44	0.41	0.099
Staging						
I + II	83	1.36	0.56	115	0.99	0.97
III + IV	487	0.92	0.45	494	1.06	0.58
Grading						
I + II	203	0.96	0.83	189	0.83	0.29
III	392	1.05	0.68	315	1.15	0.29
Chemotherapy						
Taxol + platin	356	0.88	0.4	380	0.99	0.9

Distinction was made between the subgroup’s histology (serous or endometrioid), staging (I + II or III + IV), grading (I + II or III) and chemotherapy (taxol + platin). Data were analyzed by the Kaplan–Meier–Plotter. Number of cases, HR and *p*-value are given. Statistically significant values are marked in bold typing

**Table 3:** Correlation between the expression of HYAL1-HYAL3 and the OS or PFS of ovarian cancer patients

Genes	OS			PFS		
	Cases	HR	*p*-value	Cases	HR	*p*-value
HYAL1						
Histology						
Serous	1207	0.99	0.85	1104	1.02	0.75
Endometrioid	37	0.73	0.73	51	0.81	0.65
Staging						
I + II	135	0.8	0.56	163	0.81	0.45
III + IV	1220	1.01	0.92	1081	0.99	0.91
Grading						
I + II	380	1.05	0.73	293	1.14	0.37
III	1015	1.03	0.73	837	0.91	0.26
Chemotherapy						
Taxol + platin	776	1.14	0.18	698	0.97	0.76
HYAL2						
Histology						
Serous	1207	0.91	0.24	1104	1.04	0.58
Endometrioid	37	0	**0.0038**	51	0.17	**5.00E-04**
Staging						
I + II	135	0.98	0.97	163	0.71	0.24
III + IV	1220	0.89	0.12	1081	1.07	0.36
Grading						
I + II	380	0.86	0.3	293	0.88	0.37
III	1015	0.91	0.27	837	1.03	0.7
Chemotherapy						
Taxol + platin	776	0.93	0.42	698	0.93	0.4
HYAL3						
Histology						
Serous	1207	0.83	**0.016**	1104	0.94	0.41
Endometrioid	37	0.44	0.35	51	0.67	0.4
Staging						
I + II	135	0.55	0.14	163	1.21	0.5
III + IV	1220	0.83	**0.016**	1081	0.92	0.27
Grading						
I + II	380	0.69	**0.011**	293	1.06	0.67
III	1015	0.88	0.15	837	0.92	0.32
Chemotherapy						
Taxol + platin	776	0.84	0.065	698	0.9	0.21

Distinction was made between the subgroup’s histology (serous or endometrioid), staging (I + II or III + IV), grading (I + II or III) and chemotherapy (taxol + platin). Data were analyzed by the Kaplan–Meier–Plotter. Number of cases, HR and *p*-value are given. Statistically significant values are marked in bold typing

**Table 4:** Correlation between the expression of HYAL4, PH20 and HYALP1 and the OS or PFS of ovarian cancer patients

Genes	OS			PFS		
	Cases	HR	*p*-value	Cases	HR	*p*-value
HYAL4						
Histology						
Serous	1207	0.88	0.097	1104	0.98	0.81
Endometrioid	37	0.64	0.62	51	0.81	0.67
Staging						
I + II	135	0.68	0.33	163	0.79	0.41
III + IV	1220	0.85	**0.035**	1081	0.98	0.77
Grading						
I + II	380	0.93	0.63	293	1	0.99
III	1015	0.89	0.17	837	0.98	0.82
Chemotherapy						
Taxol + platin	776	0.88	0.19	698	0.97	0.71
PH20						
Histology						
Serous	1207	1.04	0.57	1104	1.08	0.27
Endometrioid	37	0.62	0.6	51	0.3	**0.015**
Staging						
I + II	135	0.48	0.071	163	0.58	0.062
III + IV	1220	0.99	0.91	1081	1.01	0.84
Grading						
I + II	380	0.92	0.58	293	1	1
III	1015	1.06	0.47	837	1.06	0.51
Chemotherapy						
Taxol + platin	776	1.19	0.07	698	1.1	0.26

Distinction was made between the subgroup’s histology (serous or endometrioid), staging (I + II or III + IV), grading (I + II or III) and chemotherapy (taxol + platin). Data were analyzed by the Kaplan–Meier-Plotter. Number of cases, HR and p-value are given. Statistically significant values are marked in bold typing

Table 2 shows that high expression of HAS1 (HR = 1.3, *p*-value = 0.00033) had a negative impact on PFS for serous ovarian cancer patients. Furthermore, HAS2 had a negative correlation with the OS (HR = 1.26, *p*-value = 0.0027) and PFS (HR = 1.31, *p*-value = 0.00021). High expression of HYAL3 was correlated with a better OS (HR = 0.83, *p*-value = 0.016), which is shown in Table 3. It is not possible to compute a Hazard Rate in case there is no event in one of the cohorts defined by the gene expression, as the HR will be either 0 or infinite in these cases. In such cases, we adjusted the HR to < 0.1. For patients with endometrioid ovarian cancer expression of HYAL2 (HR = 0.17, *p*-value = 5.00 × 10^–4^) and PH20 (HR = 0.3, *p*-value = 0.015) was associated with better PFS (Tables 3, 4).

Referred to staging, no correlation of the HA-associated genes was found for patients in staging I + II (Table 2–4). High expression of HAS1 (HR = 1.24, *p*-value = 0.0033) was associated with worse PFS of patients in staging III + IV, as shown in Table 2. For staging III + IV HAS1 also correlated with worse OS (HR = 1.18, *p*-value = 0.03) and PFS (HR = 1.2, *p*-value = 0.012) (Table 2). A positive influence on the OS had the expression of HYAL3 (HR = 0.83, *p*-value = 0.016) and HYAL4 (HR = 0.85, *p*-value = 0.035) as shown in Tables 3 and 4.

For patients in grade I + II high expression of HAS2 showed a negative impact on the OS of ovarian cancer patients (HR = 1.44, *p*-value = 0.013). For grade III there was a worse PFS for patients with high expression of HAS1 (HR = 1.29, *p*-value = 0.0029) and HAS2 (HR = 1.29, *p*-value = 0.0027) (Table 2). High expression of HYAL3 (HR = 0.69, *p*-value = 0.011) had a positive association with the OS of patients (Table 3).

Finally, we analysed the prognostic impact of HA pathway constituents in patients related to chemotherapy treatment with the combination of taxol and cisplatin. The expression of HAS1 had a negative impact on the PFS of patients (HR = 1.25, *p*-value = 0.011) (Table 2). The expression of HAS2 was associated with worse OS (HR = 1.21, *p*-value = 0.045) and PFS (HR = 1.23, *p*-value = 0.019) (Table 2). Correlations in the context of treatment with taxol or cisplatin can be found in the supplementary information (Supplementary Table S2).

In conclusion, HAS2 appeared to be the enzyme of the HA system with the biggest impact on the survival of ovarian cancer patients. Therefore, we decided to study the functional impact of HAS2 depletion using an in vitro siRNA approach in human ovarian cancer cell lines.

### HAS2 depletion results in a moderate cell-type specific dysregulation of HYAL3

**Fig. 3:**
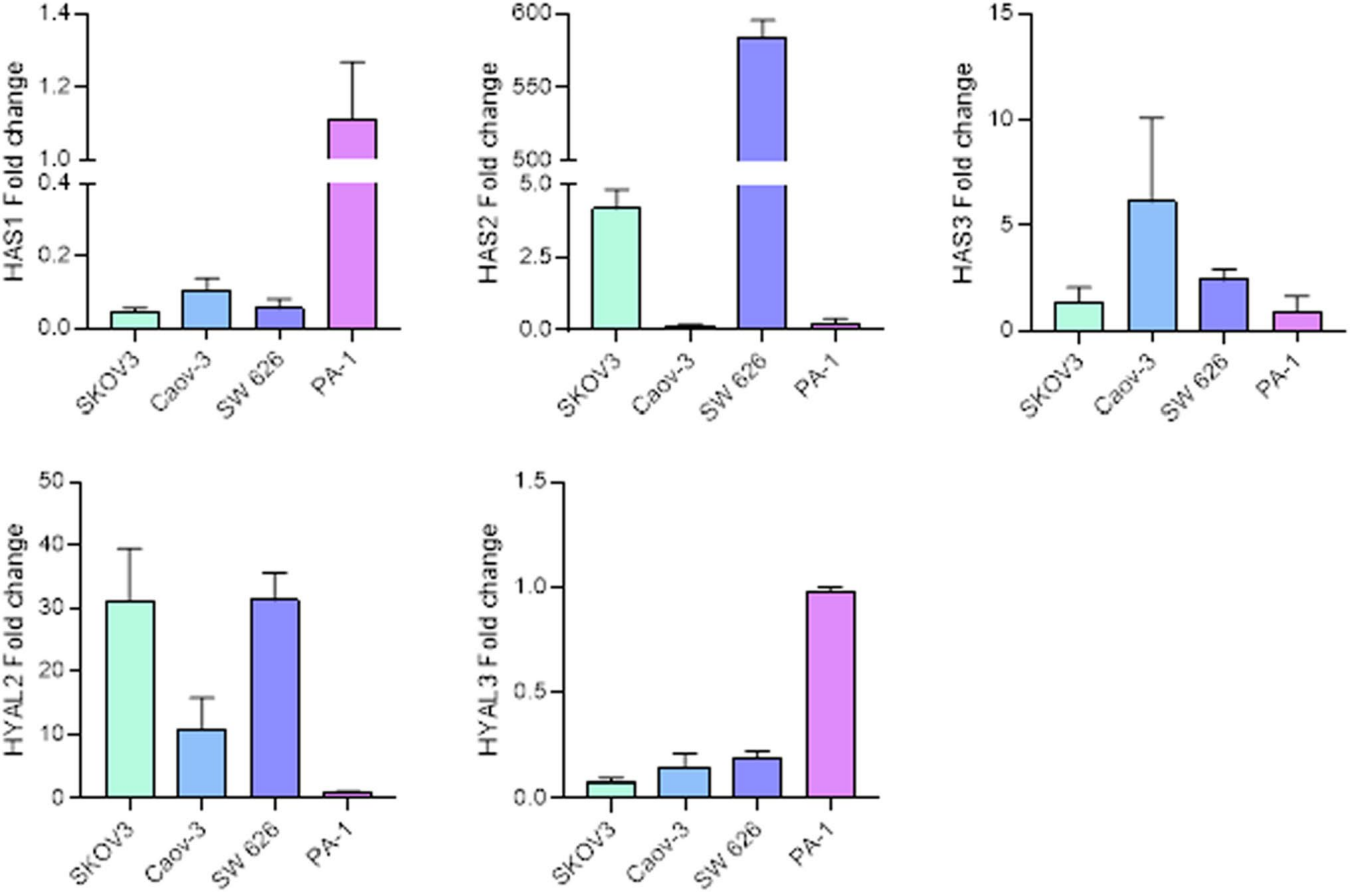
HAS1, HAS2, HAS3, HYAL2 and HYAL3 gene expression levels in SKOV3, Caov-3, SW 626 and PA-1ovarian cancer cells, as measured by qPCR. The mean value was given with the standard error. Data represent the results of 3 independent experiments with *n* = 2 or 3 independent replicates under the same conditions. The gene expression levels are shown relative to the expression in PA-1 cells, which were set to a mean value of 1

As a first step, a panel of ovarian cancer cell lines (i.e., SKOV3, Caov-3, SW 626 and PA-1) was analyzed for the expression of diverse genes involved in HA metabolism. These cell lines correspond to the ATCC ovarian cancer panel with varying degree of genetic complexity. HAS1, HAS2 and HYAL3 expression levels showed to be comparable in all adenocarcinoma cell lines SKOV-3, Caov-3 and SW 626 cells, whereas the teratocarcinoma cell line PA-1 cells expressed higher levels of all the three genes (Figs. 3a, b, e). As for HAS3, the expression was slightly higher in Caov-3 cells, with respect to all the other cell lines (Fig. 3c). HYAL2 expression was comparable in SKOV3 and SW 626 and substantially higher with respect to Caov-3 and PA-1 cells (Fig. 3d). Since SKOV3 and SW 626 displayed very similar gene expression profiles related to HA metabolism and showed the highest HAS2 expression in this panel, we decided to focus on these lines for our subsequent experiments.

**Fig. 4:**
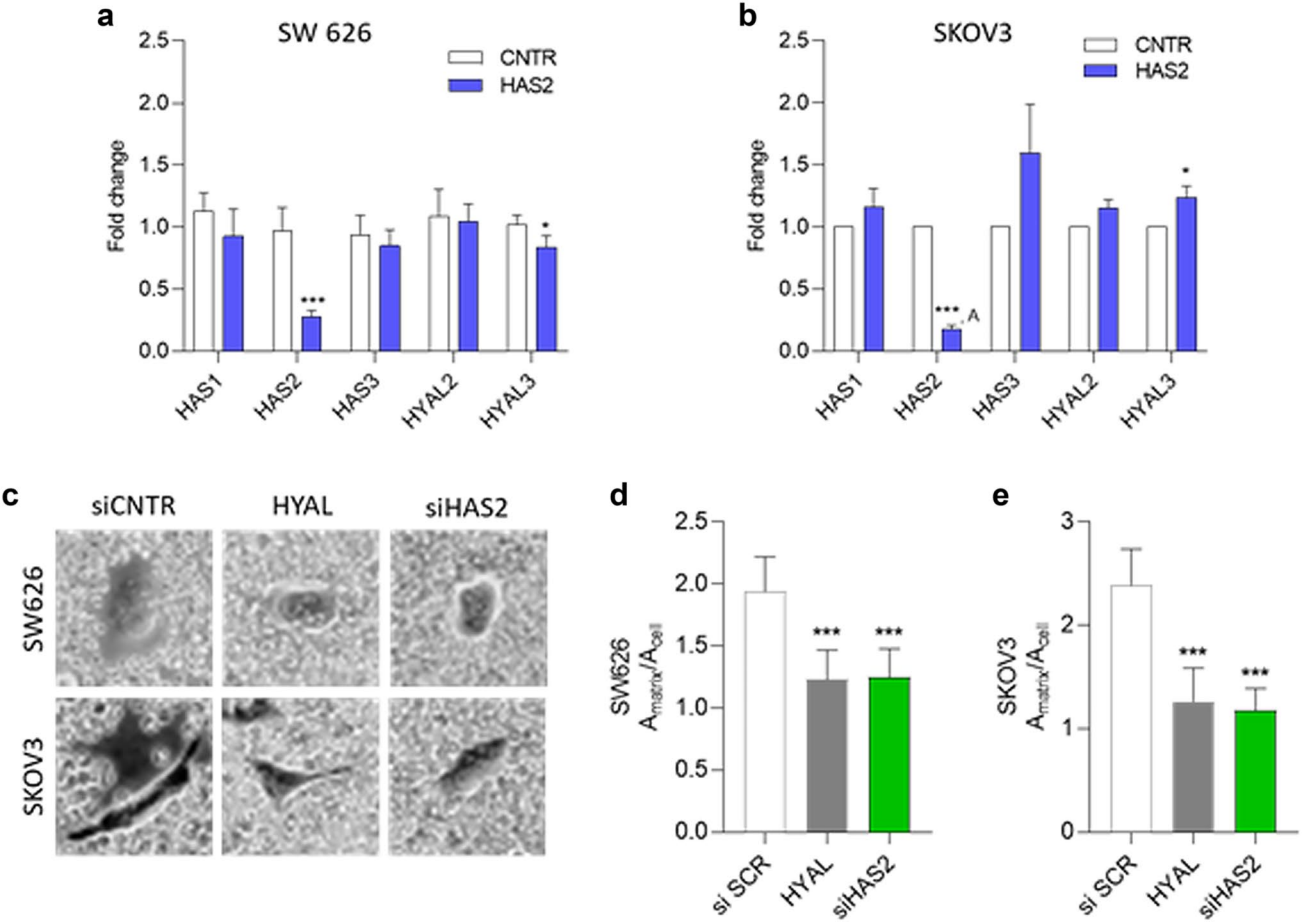
Impact of HAS2 knockdown and its influence on HA production and the expression of HAS1, HAS3, HYAL2 and HYAL3 in SW 626 and SKOV3 ovarian cancer cells. **a**, **b** qPCR confirmation of HAS2 knockdown and impact on the expression of HA-related genes. The mean value is given with the standard error. Data represent the results of 4 independent experiments with *n* = 2 or 3 independent replicates under the same conditions. **a** The mean value was calculated out of 7 values, in 6 HAS2 knockdown samples the HAS2 expression levels were under the limit of detection. **c** Representative images of particle exclusion assay of SW 626 and SKOV3 pericellular space as readout of HA production. Cells were transfected for 24 h with 20 nM siRNA against HAS2 or scrambled control siRNA or treated with 2 U/ml *Streptomyces hyalurolyticus* hyaluronidase. **d**, **e** Quantification of HA pericellular matrix for SW 626 and SKOV3 cell lines. Data are shown as mean ± SEM of three independent experiments. Results are expressed as the ratio between the area of ECM delimited by red blood cells and the area of the cell by using ImageJ software. *p ≤ 0.05, ***p ≤ 0.0001

At this point, we first asked whether HAS2 knockdown affects the expression of other enzymes of the HA system. For this purpose, we used siRNA transfection and qPCR to detect the HAS2 knockdown and to compare the expression of HAS1, HAS3, HYAL2 and HYAL3 in HAS2 knockdown cells with the expression in control cells. These enzymes were chosen because they showed the greatest impact on patient survival in the Kaplan–Meier-Plotter. It was shown that the knockdown of HAS2 resulted in a significant (*p* < 0.001) and substantial downregulation (> 75%) of its expression in both SKOV3 and SW 626 cell lines (Fig. 4a, b, Supplementary Figure S2). No HAS2 expression was detectable in 6 samples of SKOV3 cells in the HAS2 knockdown group, suggesting that the expression rate was below the detection limit. While these data speak for a successful knockdown, these values were not included in the calculation. Evaluation of the kinetics of HAS2 knockdown revealed that HAS2 mRNA levels were substantially and significantly downregulated by > 75% for 24 h and 48 h after knockdown, returning to basal levels 4d–7d after transient transfection (Supplementary Figure S2). The measurement of pericellular HA confirmed that both SW 626 and SKOV3 cells had significantly lower amounts of pericellular HA upon HAS2 silencing with respect to control cells (Fig. 4c, d). The HAS2 knockdown did not have a significant influence on the expression of HAS1, HAS3 and HYAL2. HYAL3 was marginally downregulated by about 20% in SW 626 cells (*p* < 0.05), whereas it was upregulated to a similar extent in SKOV3 cells (*p* < 0.05) (Fig. 4a, b). mRNA levels of the HA receptors CD44 and RHAMM, and the HA binding proteoglycan versican (VCAN) were not changed upon HAS2 silencing with respect to control in both cell lines (Supplemental information, Figure S3).

Furthermore, the influence of HAS2 knockdown on the expression of HAS1, HAS3, HYAL2 and HYAL3 in cells treated with chemotherapy was investigated. A distinction was made between therapy with taxol, cisplatin and the combination of taxol and cisplatin. With all therapies, no significant difference was found with regard to the expression of HAS1, HAS3, HYAL2 and HYAL3. More detailed data are provided in the Supplementary Figure S4. In conclusion, we could prove a successful HAS2 knockdown in SKOV3 and SW 626 cells. As a result, HYAL3 was moderately, yet significantly dysregulated in a cell-type specific manner.

### HAS2 knockdown affects the formation of tumour cell spheroids

**Fig. 5:**
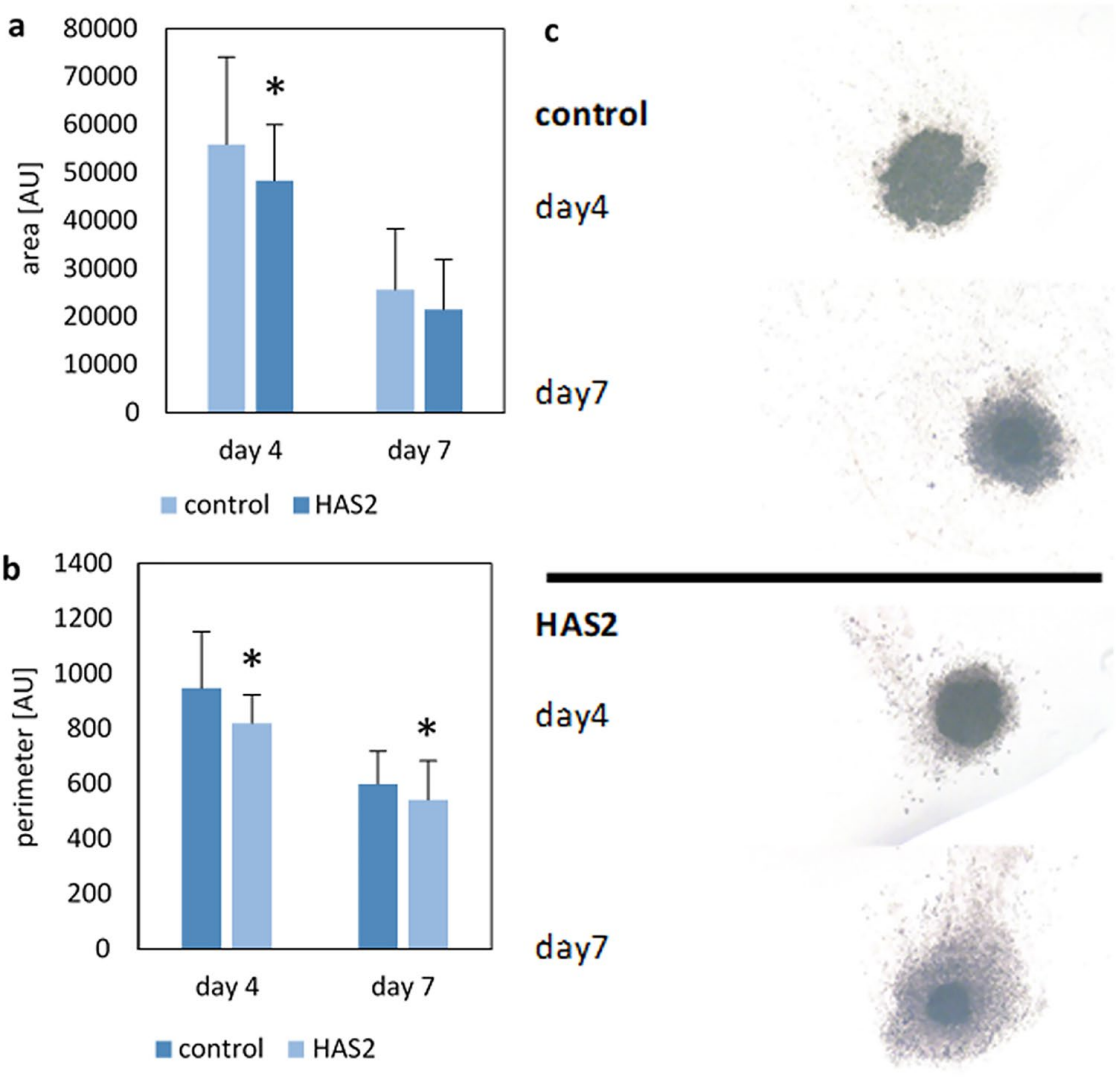
Impact of HAS2-depletion on the sphere formation capacity of SKOV3 cells. Hanging drop method was used to show differences in cell cohesion and sphere formation capability of HAS2 knockdown SKOV3 cells compared to control SKOV3 cells. **a**, **b** Area and perimeter of the spheres excluding the diffuse edge. The area or perimeter of spheres of HAS2 knockdown and control cells was measured at day 4 and day 7. *p ≤ 0.05, **c** Representative pictures of spheres in drops of HAS2 knockdown cells and control cells. Note presence of a solid dark core and a light, diffuse edge. The values were built out of data of 4 experiments á 12 drops for HAS2 knockdown and control cells (*n* = 48). *AU *arbitrary unit

HA is an important factor that influences cell cohesion and stability. Moreover, a role for HA in cancer stem cell function has been described (D. Vitale et al. 2019a, b). To test the possible influence of HAS2 knockdown on the capability of ovarian cancer cells to form tumour spheroids, a hanging drop assay was performed. In the hanging drop method, these factors were represented by the size of the area and the perimeter of cell spheres in each drop. Differences were between control and HAS2 siRNA treated cells were analyzed regarding the area and perimeter of the spheres and area and perimeter of the spheres plus a diffuse edge/margin that was visible under some of the treatment conditions. Spheroids of control SKOV3 cells and HAS2 knockdown cells on day 4 and day 7 are shown in Fig. 5c We found that the area of spheres of HAS2 knockdown cells was significantly smaller on day 4 (*p*-value = 0.017) (Fig. 5a). Furthermore, the perimeter of these was smaller for HAS2 knockdown cells compared to control cells on both days (day 4 *p*-value = 2.72 × 10^–4^, day 7 *p*-value = 0.035) (Fig. 5b). For values including the diffuse edge, the area of HAS2 knockdown cells was significantly higher on day 4 (*p*-value = 0.047) and day 7 (*p*-value = 4.97 × 10^–10^) (Supplementary Figure S5a). Referred to the perimeter, measured values were significantly higher for HAS2 knockdown cells on day 7 (*p*-value = 4.25 × 10^–5^) (Supplementary Figure S5b). Besides this, we observed that a diffuse edge was formed in 25% of the drops with control cells on day 4 and in 54% on day7 (Supplementary Figure S5c). Compared to this HAS2 knockdown cells formed a diffuse edge in 76% of the drops on day 4 and 100% on day 7 (Supplementary Figure S5c). In the evaluation of the diffuse edge, only the drops that formed an edge were included. Two drops of control cells on day 4 and 4 drops on day 7 did not form spheroids. HAS2 knockdown cells did not form a spheroid in 2 drops for both days. These samples were not included in the results. SW 626 cells failed to form proper spheroids in the hanging drop assay, as only loose cell aggregates were seen, precluding an analysis of HAS2 depletion in this assay (Supplementary Figure S6). To conclude, we found out that HAS2 knockdown SKOV3 cells formed significant smaller spheres with bigger edges, especially on day 7. Furthermore, knockdown SKOV3 cells formed this edge more often.

### Impact of HAS2 siRNA depletion on cell viability and the response to chemotherapy

**Fig. 6:**
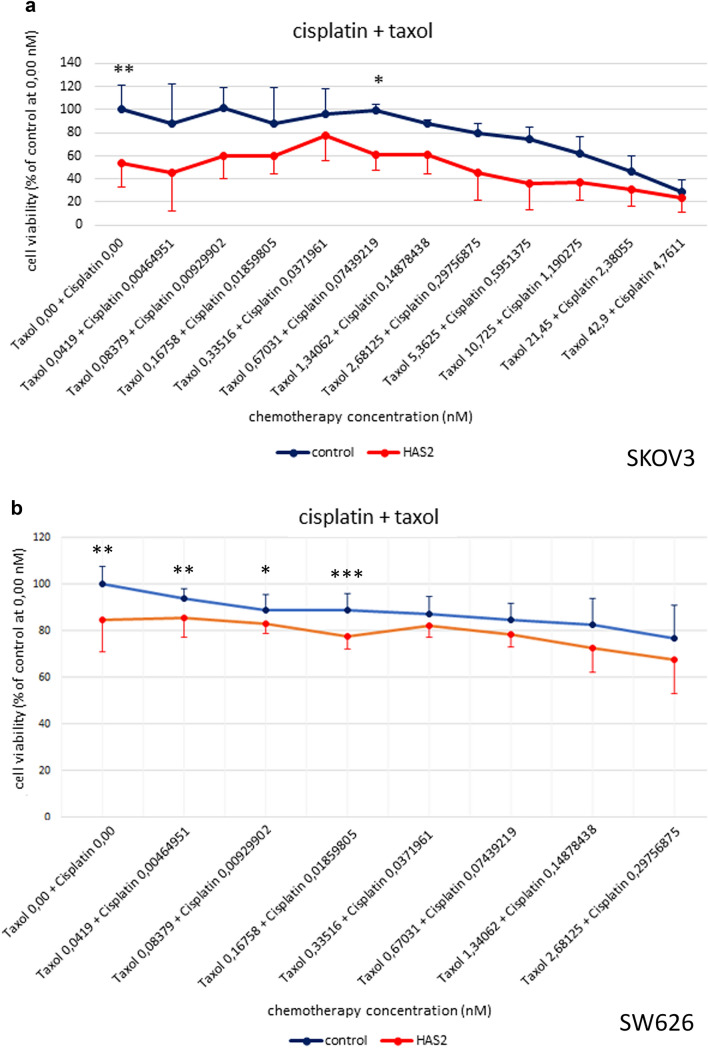
Viability of HAS2 knockdown and control ovarian cancer cells at different concentrations of taxol and cisplatin, measured by MTT assay. **a** SKOV3 cells, **b** SW 626 cells. All values are given in % based on the concentration of control cells at 0.00 nM chemotherapy treatment. Results represent mean value ± SD for 3 experiments under same conditions. *p ≤ 0.05, **p ≤ 0.001

As our Kaplan–Meier-Plotter analysed had indicated an impact of HAS2 on OS and PFS of patients with prior chemotherapy treatment, we analysed the impact of HAS2-depletion in SKOV3 cells subjected to different concentrations of chemotherapy in vitro. The MTT Assay was used as a well-established and robust assay (Sargent 2003) to assess whether the ovarian cancer cell viability is influenced by HAS2 knockdown. In all chemotherapy treatment conditions, 8–11 serial 1:2 dilutions of the combinatorial treatment with taxol and cisplatin were applied to control cells and HAS2 knockdown cells (Fig. 6).

Starting with the lowest concentration of chemotherapy the therapeutics had a concentration of 0.00 nM. The viability of HAS2 knockdown SKOV3 cells under these basal conditions was 46.68% lower compared to control cells (*p*-value = 0.0002) (Fig. 6a). A significant value was measured at 0.6703 nM taxol and 0.0744 nM cisplatin. The viability of HAS2 knockdown SKOV3 cells was 38.7% smaller (*p*-value = 0.022) (Fig. 6a). Similar effects were seen in SW 626 cells, where HAS2 knockdown resulted in a significant decrease in cell viability, albeit to a lesser extent (16%, p < 0.01) compared to SKOV3 cells (Fig. 6b). The significant difference in viability of HAS2 knockdown and control SW 626 cells persisted upon treatment with 0.16758 nM taxol and 0.0186 nM cisplatin. All in all, in both cell lines tested, the viability of HAS2 knockdown cells was lower than control cells, indicating that the impact of HAS2 depletion alone on cell viability was higher than a possible effect of HAS2 on the chemotherapy response under our assay conditions. The effects on SKOV3 cell viability of taxol or cisplatin treatment alone are shown in the supplementary information (Supplementary Figure S7).

Finally, we evaluated if HAS2 depletion may affect the migratory capacity of ovarian cancer cells, employing a scratch wound assay. No significant impact on SKOV3 migration was observed upon HAS2 knockdown (Supplementary Figure S8). SW 626 cells detached as cell sheets at the scratch wound margins, precluding meaningful quantitative analysis in this assay (data not shown).

### String analysis reveals the interconnection of the HA system and pathogenetic factors in ovarian cancer

**Fig. 7:**
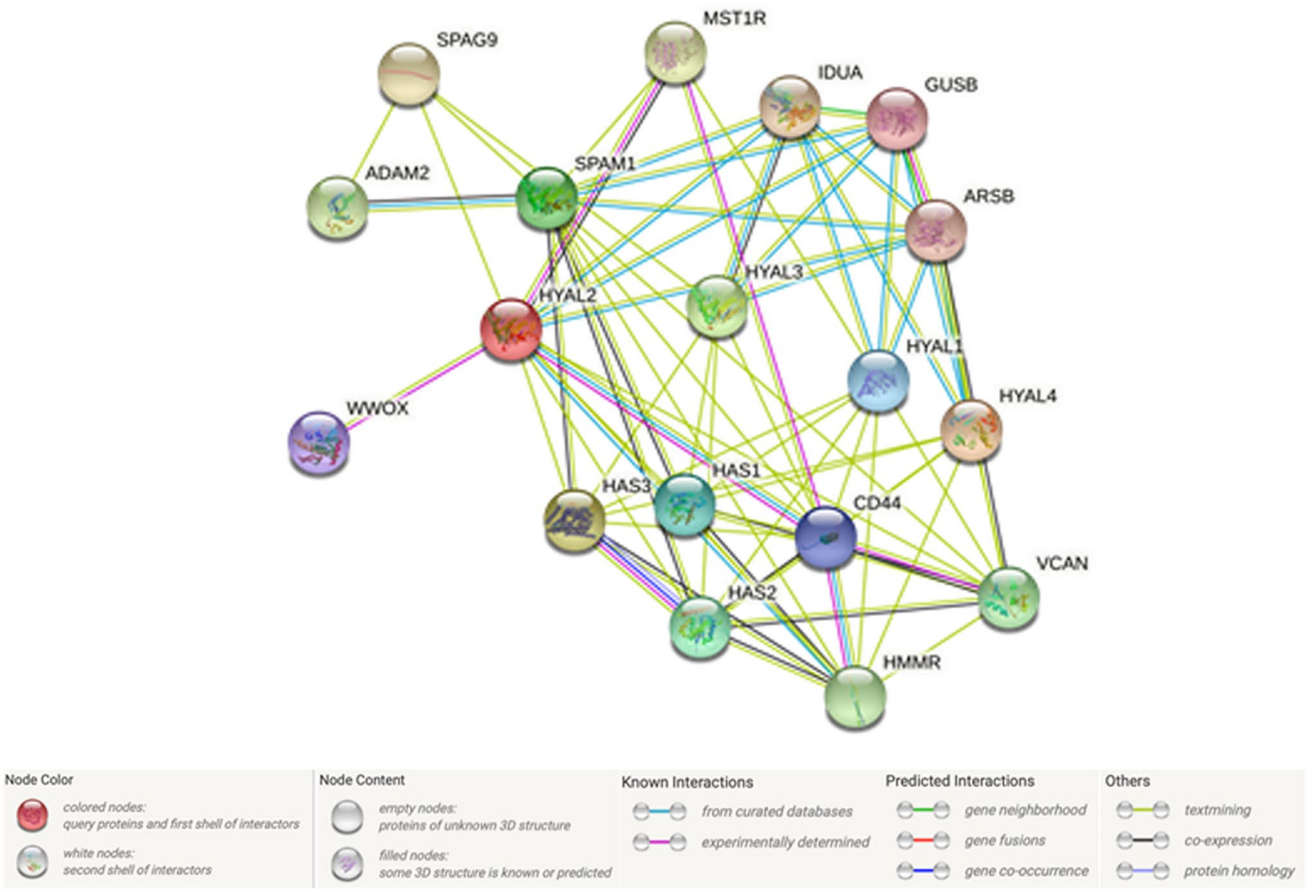
STRING analysis for protein–protein interactions of HA pathway constituents. With the use of STRING database (https://string-db.org) the interactions of the proteins, analyzed in this study, are shown. Medium confidence threshold of 0.004

Our last step of the analysis was the use of the STRING tool to show interactions of HAS1-3 and HYAL1-5 (Fig. 7) between each other and the 10 closest interactions with other proteins. For each protein, the interactions were analyzed related to gene neighbourhood, gene fusions, gene co-occurrence, experimentally determination, curated databases, co-expression, protein homology and text mining. HYALP1 was not analyzed by the STRING tool. Referred to the HA system it was shown that there is high interaction between HAS2 and HAS3. HAS1-3 interacted with PH20 due to co-expression and text mining. Text mining indicated an interaction between HAS1-3 and HYAL2 and HYAL3 and between HAS2 and HYAL4 (Fig. 7).

Related to other proteins, especially HAS2 and HAS3 showed high interaction with UGDH, CD44 and HMMR. Besides this, HAS1 and HAS2 cooperated with VCAN. In addition, a strong interaction between PH20 and ADAM2 was evident (Fig. 7).

For HYALs, a high interaction was found between HYAL2 and CD44, HMMR, Macrophage stimulating 1-receptor (MST1R) and WWOX. PH20 showed high interaction with ADAM2 (Fig. 7).

The other proteins shown in Fig. 7 are interacting in less relevant size. Therefore, they were not named in detail.

## Discussion

In this study, we addressed the question if the level of expression of HAS1-3, HYAL1-5 and HYALP1 has an influence on the survival of ovarian cancer patients. We hypothesized that the expression of HAS2 could have an effect on the success of therapy and sphere formation capability and cohesion of the tumour cells.

Our TNMplot analysis of over 700 ovarian cancer specimens revealed that HAS1, HYAL1 and HYAL4 mRNA expression is significantly upregulated, whereas HAS2, HYAL2 and HYAL3 mRNA expression is significantly downregulated in ovarian cancer tissue compared to controls. These data underscore the clinicopathological significance of the HA biosynthetic and degradative system in ovarian cancer. Of particular relevance, our experiments highlighted that the expression of HAS1 and especially the expression of HAS2 correlates with poorer survival of ovarian cancer patients. Considering the strong association between ovarian cancer aggressiveness and HA deposition (Anttila et al. 2000), we knocked down HAS2 to evaluate if its expression correlates with ovarian cancer cell lines' aggressiveness. HAS2 knockdown has little effect on the expression of HASs and HYALs, with a small increase in HYAL3 expression. Interestingly, HAS2 appears to have an influence on cell cohesion capacity, which was significantly lower with HAS2 deletion, as shown by smaller spheroids in the hanging drop method. Moreover, cell viability was significantly reduced by HAS2 knockdown, but it was only moderately reduced in response to chemotherapy in both controls and HAS2 knockdown cells. All in all HAS2 expression goes along with lower OS and PFS of ovarian cancer patients, and, indeed, our experiments suggest that HAS2 seems to be important for stimulating tumour and cell growth and stability and it encourages cell viability.

### Influence of hyaluronan synthases HAS1-3 on ovarian cancer and patient’s survival

First, we compared the survival time of patients with low expression of HA-related enzymes with those with high expression by the use of the Kaplan–Meier Plotter. We found that HAS1 and HAS2 expression correlated with worse survival of ovarian cancer patients. However, in some subgroups, the data must be interpreted with caution, especially when the number of cases was below 50. This applies to HAS1 and HAS2 to data of OS of patients with endometrioid ovarian cancer and of PFS of patients in grade 1. For HAS3 it concerns OS and PFS of patients with endometrioid ovarian cancer and OS and PFS of patients in grading I. Due to the small number of cases, false tendencies could arise.

We analysed a panel of four different ovarian cancer cell lines (i.e., SW 626, SKOV3, Caov-3 and PA-1) and measured the expression levels of several HA-related genes, namely HAS1-3 and HYAL2-3. As SW 626 and SKOV3 had comparable gene expression profiles and the highest relative expression of HAS2, these two cell lines were chosen for the subsequent characterizations.

The influence of HAS1 on the survival of ovarian cancer patients has not yet been studied in detail. However, it has been shown that high HAS1 expression is associated with poor patient survival for ovarian cancer, colon cancer, Waldenström´s macroglobulinemia and multiple myeloma (Siiskonen et al. 2015), and its downregulation correlates with lower growth and development of bladder cancer due to lower hyaluronan production (Golshani et al. 2008). Our results from the Kaplan Meier-Plotter confirm that, indeed, high HAS1 expression leads to lower survival of ovarian cancer patients. One assumption is that high HAS1 expression ends in higher HA production. This could lead to greater tumour growth. This has already been shown for the ability of prostate cancer to metastasise to the bone marrow (Simpson et al. 2002, 2001).

Among all HASs, HAS2 is the most important one implied in both physiological and pathological conditions, including cancer (Camenisch et al. 2000; Passi et al. 2019). Our results report that the higher the HAS2 expression, the lower the OS and PFS for ovarian cancer patients. This finding is in accordance with the literature stating that high HAS2 expression also leads to short OS in pancreatic cancer patients (Yu et al. 2021). Moreover, elevated HAS2 expression is also found in breast cancer cell lines compared to normal breast tissue, and its knockdown leads to decreased proliferation and increased apoptosis (Li et al. 2015). Finally, a correlation between high coexpression of HAS2 and HYAL1 and strong tumour growth and angiogenesis was observed for prostate carcinoma (Simpson 2006).

Our results on the ability of SKOV3 ovarian cancer cell lines to form spheroids confirmed that, effectively, HAS2 could be involved in the reduction of tumour aggressiveness, as we observed a significantly poorer cell cohesion in HAS2 knockdown cells with respect to control cells. A possible explanation could be related to the significant reduction of HA synthesis as a result of HAS2 silencing, which we demonstrated via the particle exclusion assay.

This hypothesis is supported by a study reporting that high expression of HAS and HA correlates with higher metastasization and invasiveness in different tumour types (Jojovic et al. 2002). Furthermore, it has also been shown for ovarian clear cell carcinomas that tumour cell growth is inhibited by low levels of HA (Kato et al. 2016). Concomitantly, we also found significantly reduced cell viability in HAS2 knockdown cells, even without prior chemotherapy treatment. This could be an indication that the cells grow worse due to a lack of HA production. This also fits with the statements of Okuda and colleagues that high HAS2 expression in breast cancer cells correlates with increased growth and metastasis than control cells. Furthermore, it indicates a lower OS time for patients (Okuda et al. 2012). A caveat is associated with our transient transfection approach, as our qPCR analysis revealed that the knockdown was not stable for extended periods. Particularly for the spheroid formation the assay times exceeded the knockdown duration at later timepoints, suggesting that heterogeneity in the cell population may have contributed to the phenotype and acted as a confounder. Nevertheless, this heterogeneity has resulted from HAS2 knockdown, highlighting a causative relation to HAS2.

Interestingly, HAS2 knockdown and control cells both showed a moderate response to the different chemotherapy treatments. The therapeutic effect of chemotherapy for ovarian cancer can be low due to their intrinsic chemotherapy resistance (Ricciardelli et al. 2013). We demonstrated that this is not significantly changed by HAS2 knockdown, but the viability of HAS2 knockdown cells is fundamentally poorer. Indeed, more successful therapy for chemotherapy-resistant serous ovarian cancer cells seems to be possible through the combination of carboplatin and 4-methylumbelliferone (4-MU). This inhibits HA production, cell survival and spheroid formation in these cells. This is therapeutically significant, as increased HAS2 and HAS3 expression was observed in chemotherapy-resistant ovarian cancer cells (Lokman et al. 2019). In addition, Bourguignon et al. showed that chemotherapy resistance in ovarian and breast cancer cells arises via the HA–CD44 interaction by inducing the binding of Ankyrin to MDR1 (Bourguignon et al. 2008, p 44). Ricciardelli et al. also showed that the HA–CD44 signalling pathway could be an important approach for treating the development of resistance to carboplatin in ovarian cancer patients. Indeed, after carboplatin treatment, the expression of HAS2, HAS3, ABCC2 and HA secretion increased. A high HA level in turn correlated with higher survival of CD44 positive ovarian cancer cells. HA thus appears to be a relevant factor in relation to the high survival of tumour cells after carboplatin treatment (Ricciardelli et al. 2013). In order to be able to treat ovarian cancer optimally, further research is needed in this area.

The fact that low expression of HAS2 is correlated with lower tumour cell growth is strengthened by the results of String analysis that HAS1-3 interact close with UGDH. UGDH plays a role in glycosaminoglycan synthesis and therefore is also important in relation to ECM and the synthesis of HA (Egger et al. 2011). CD44 is a non-kinase transmembrane proteoglycan, which mainly ligand is HA. RHAMM also has HA as its main ligand. The binding of HA to CD44 or RHAMM allows intracellular adapter molecules to bind. This promotes cell adhesion, cell migration and cell proliferation (Chen et al. 2018; Savani et al. 2001). Notably, we also found an interaction between HAS1-2 and VCAN, which is an essential proteoglycan supporting growth, survival, angiogenesis, metastasis, migration and invasion of tumour cells (Li et al. 2020; Fujii et al. 2015).

In a study from 2003, it was found that HAS3 is overexpressed in metastatic tissue of colon carcinoma. Furthermore HAS3 knockdown showed inhibition in the growth of both colon cancer and oesophageal squamous cell carcinoma cell lines (Bullard et al. 2003; Twarock et al. 2011). However, we did not find a significant correlation between HAS3 expression and ovarian cancer patient survival. Furthermore, there was no significant connection between HAS2 knockdown and HAS3 expression although the string analysis showed a strong correlation (Fig. 5A). In summary, HAS3 did not appear to play a central role in the survival of ovarian cancer patients in our study.

A deep investigation into the molecular mechanism by which elevated HA deposition drives ovarian cancer aggressiveness is essential to try and develop an efficient targeted therapy aimed at lowering the overall HA amount in the tumor stroma. At the time being, a few molecules have been investigated to target and block HA synthesis and/or signaling. 4-MU is a well known inhibitor of HA synthesis, which is already used in the clinics for the treatment of biliary spasms (Abate et al. 2001). Its potential beneficial effect in the treatment of several cancers such as breast, pancreatic and skin cancers, have been investigated—all studies reports that 4-MU can inhibit the proliferation, migration, and invasion of multiple cancer cells, both in vitro and in vivo (Urakawa et al. 2012; Edward et al. 2010; Hajime et al. 2007; Morohashi et al. 2006). However, the potential long-term consequences of 4-MU administrations are still under debate, as generalised inhibition of HA synthesis could lead to diverse side effect, among which the worsening of atherosclerosis observed in Apo-E deficient mice (Nagy et al. 2010).

A peptide-based aproach targeting CD44 and RHAMM is ongoing (Hauser-Kawaguchi et al. 2019; Weng et al. 2022). Particularly, the A6 eight-aminoacid peptide binds to CD44, enhancing HA binding and the downstream phosphorylation of CD44 signalling components, such as focal adhesion kinase (FAK) and Mitogen-activated protein kinase kinase (MEK). Even though behaving as a CD44 agonist, A6 treatment reduced the migration of cancer cells in vitro and demonstrated increased progression-free survival in patients with ovarian cancer with a positive safety profile (Gold et al. 2012). Preclinical studies using anti-CD44 antibodies to treat cancer have shown promising results, yet failing the clinical trials examining the safety and efficacy of anti-CD44 therapies (Xu et al. 2020).

Overall, our results and already known publications indicate that increased HAS synthesis and consequently increased HA production led to increased tumour cell growth and reduced survival time, respectively. In contrast to this is the observation that high HA production is associated with lower adhesion to the peritoneum in ovarian cancer cells and therefore seems to be protective with respect to metastasis to the peritoneum. (Tamada et al. 2012) Furthermore, HA could also be used in tumour therapy for ovarian cancer patients in the form of cross-linked HA gel. This gel seems to stop further tumour growth by inhibiting the migration and proliferation of cells, as well as reducing the occurrence of adhesions (Pang et al. 2018). With regard to patients with chemotherapy-induced primary ovarian insufficiency, it has been shown in experiments with rats that HA appears to have a preventive effect in these patients due to the promotion of granulosa cells and upregulation of PGRMC1 expression (Zhao et al. 2015). These results show that HA seems to have both, positive and negative effects on ovarian cancer progression and ovarian diseases.

### Influence of HYAL1-5 and HYALP1 on ovarian cancer and the patient’s survival

With the use of the Kaplan–Meier-Plotter we could show that patients with high HYAL2 and HYAL3 expression had better survival. HYAL4 had a positive influence on patients in staging III + IV for OS and PH20 for patients with an endometrioid carcinoma for PFS. Referred to HYAL1 and HYALP1 no correlation was found. In agreement with that, it has been reported that HYAL1 is upregulated in clear cell and mucinous ovarian cancer cells, but not in serous and endometrioid ones (Yoffou et al. 2011). Nevertheless, another group found significantly lower levels of HYAL1 in serous ovarian cancer cells. They did not find a changed regulation of HAS1-3 (Nykopp et al. 2009). With regard to the HYALs, we found most significances for HYAL2 and HYAL3. Therefore, only these two HYALs were included in the more detailed laboratory investigation. In particular, HAS2 knockdown leads to a significant upregulation of HYAL3 in SKOV3 and downregulation in SW 626 cell line. HYAL2, instead, did not show a significant correlation. The significance of the upregulation and downregulation of HYAL3 in the two cell lines should be viewed with caution—a fold change of 1.2381 and 0.8384, respectively, represents only a small change in HYAL3 mRNA levels. Whether this is a side effect or a clinically relevant result cannot be said on the basis of the qPCR results. This connection would have to be analysed in more detail to be able to draw conclusions from it. Until then, no significant correlation was found in previous studies. In contrast to our qPCR results, it was reported for breast cancer that HAS2 knockdown in Hs578T cancer cells leads to an upregulation of HAS1, HAS3 and HYAL1. Furthermore produced HA was smaller and the migration of cancer cells was slower (Li et al. 2007). Besides this, it has been reported that HAS2 knockdown in breast cancer cells leads to a downregulation of HYAL2 and CD44 (Udabage et al. 2005). One possibility for an optimized future therapy of ovarian cancer could be the treatment with Irinotecan conjugated to HA, which has been tested in mice. This could make a regionally specified therapy for ovarian cancer cells possible (Montagner et al. 2015).

The ability of HYALs in generating small HA fragments that could have a protumorigenic role makes them an appealing choice for pharmacological targeting in chemotherapy. Interestingly, several clinical trials are ongoing to study the combined use of recombinant HYALs, such as PEGPH20 (PEGylated recombinant human hyaluronidase PH20), to sensitise solid tumours to conventional chemotherapy. These recombinant HYALs have shown to reduce the HA amount in tumour stroma, thus reducing interstitial pressure and allowing the drugs to reach tumour cells and induce cell death (Provenzano et al. 2012). However, several concerns are arising from the potential adverse effects that residual small HA fragments produced by the recombinant HYALs enzymatic activity on surviving tumour cell proliferation, growth and motility.

## Conclusions

Our work is a comprehensive analysis of the correlation between all the most important HA-related genes and the aggressiveness of ovarian cancer. In summary, HAS2 may be an important prognostic factor in ovarian cancer. We could show that HAS2 expression correlates with higher tumour cell growth and viability and lower patients’ survival. Instead, a low HAS1 and HAS2 levels are associated with better patients’ survival. This also applies to the high expression of HYAL2 and HYAL3. Nevertheless, further research is needed on the relevance of the HA system in ovarian cancer. In particular, an optimization of the therapy treatment is a central research goal. In this respect, HAS2 does not seem to play a central role with regard to the sensitivity of ovarian cancer cells to the chemotherapies taxol and cisplatin. In addition, it could be researched more closely, if the possible connection between the expression of HAS2 and HYAL3 has a consequence for example for HA production or tumour cell behaviour.

## Data Availability

The datasets generated and analysed during the first part of the current study, the survival analysis and gene expression analysis, are available in the Kaplan–Meier Plotter database https://kmplot.com/analysis/ which also contains a link to the TNMplot tool. The datasets generated and analysed during the second part of the current study, the cell line data, are available from the corresponding author on reasonable request.

## Abbreviations

ECM: Extracellular matrix
HA: Hyaluronan
HAS: Hyaluronan synthase
HR: Hazard ratio
HYAL: Hyaluronidase
OS: Overall survival
PFS: Progression free survival
qPCR: Quantitative real time PCR
