# Supplementary material for: The hyaluronan-related genes HAS2, HYAL1-4, PH20 and HYALP1 are associated with prognosis, cell viability and spheroid formation capacity in ovarian cancer
Source: J Cancer Res Clin Oncol. 2022 Jun 29;148(12):3399–419. doi: 10.1007/s00432-022-04127-6 (PMC9587083; doi:10.1007/s00432-022-04127-6)
Supplement: Supplementary file 1 [file 432_2022_4127_MOESM1_ESM.docx]

Supplementary information

The hyaluronan-related genes HAS2, HYAL1-4, PH20 and HYALP1 are associated with prognosis, cell viability and spheroid formation capacity in ovarian cancer

Jette Riecks, Arianna Parnigoni, Balázs Győrffy, Ludwig Kiesel, Alberto Passi, Davide Vigetti, Martin Götte

	normal	high	medium	low	n.d.	Antibody, cases = n
HAS1	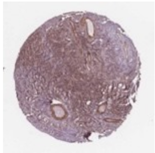	0<br>/	2<br>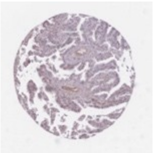	3<br>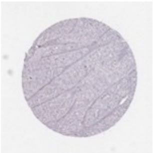	7<br>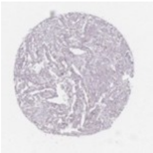	Antibody<br>HPA067602<br>12
HAS3	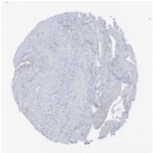	0<br>/	0<br>/	1<br>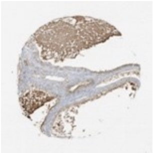	10<br>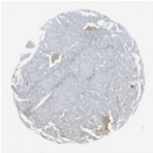	Antibody<br>CAB033850<br>11
HYAL1	not detected<br>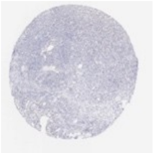	0<br>/	2<br>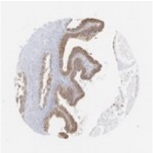	1<br>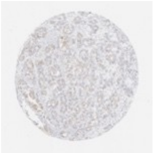	9<br>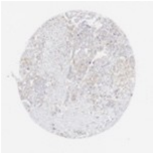	Antibody<br>HPA002112<br>12
HYAL2	not detected<br>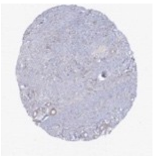	0<br>/	3<br>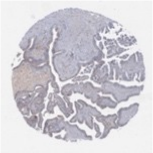	0<br>/	8<br>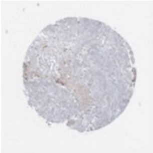	Antibody<br>HPA036436<br>11
HYAL3	n.a.<br>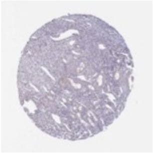	0<br>/	2<br>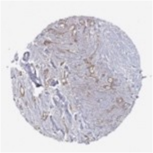	1<br>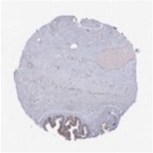	8<br>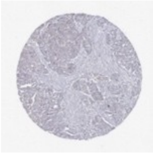	Antibody<br>HPA049402<br>11
HYAL4	n.a.<br>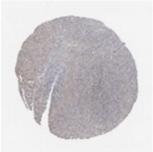	0<br>/	3<br>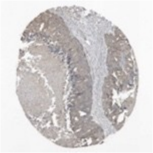	8<br>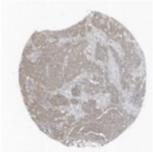	0<br>/	Antibody<br>HPA029453<br>11
PH20	not detected<br>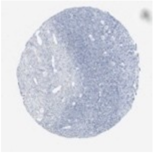	0<br>/	0<br>/	0<br>/	12<br>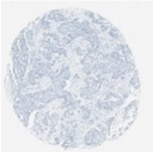	Antibody<br>HPA017984<br>12

Supplementary Figure S1. *The protein expression of HAS1, HAS3 and HYAL1-HYAL5 in ovarian tumour tissue compared to normal tissue as assessed by immunohistochemistry. The data are derived from the Human Protein Atlas (https://www.proteinatlas.org, accessed 24.02.2022). The expression in tumour tissue was categorized into high, medium, low and undetectable. Data for HAS2 protein expression were not available.*

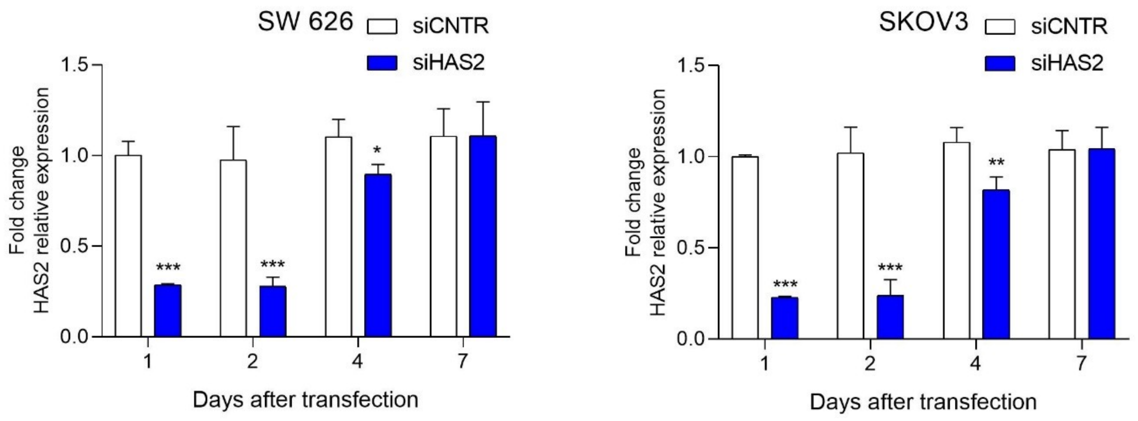

Supplementary Figure S2. *Kinetics of HAS2 knockdown stability in a 1-7d time-course as measured by qPCR HAS2 is substantially and significantly downregulated following HAS2 siRNA treatment 24h and 48h after knockdown, slowly returning to control levels after 7d. The mean value is given with the standard error. Data represent the results of four independent experiments (n = 2 or 3) under the same conditions. *p ≤ 0.05, ** p ≤ 0.01, ***p ≤ 0.001*

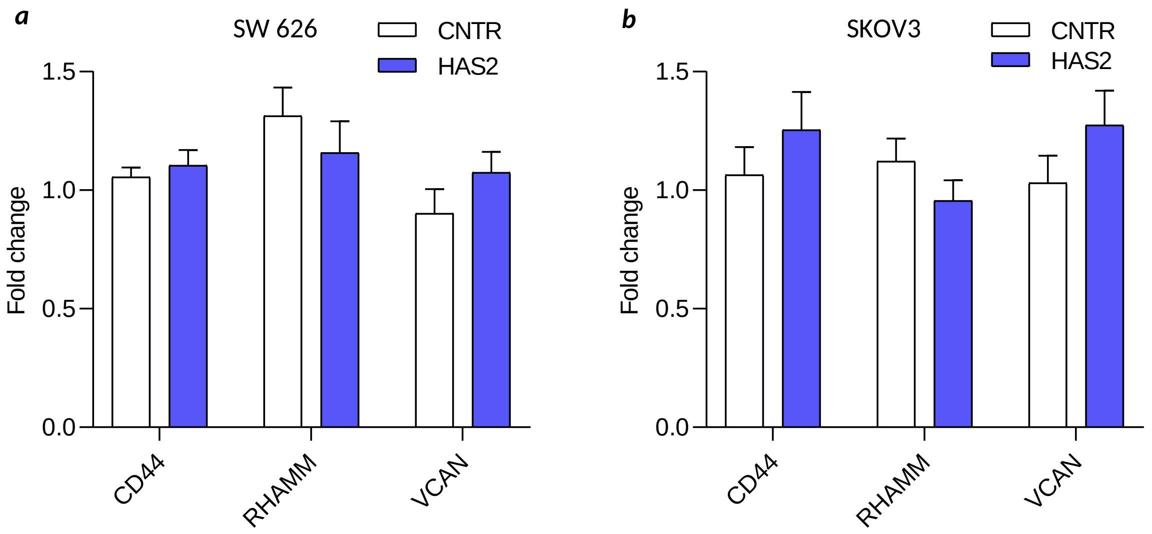

Supplementary Figure S3 *Impact of HAS2 knockdown and its influence on the expression of CD44, RHAMM and VCAN in SW 626 and SKOV3 ovarian cancer cells, as measured by qPCR. No significant changes were observed between HAS2-silenced and control samples. The mean value is given with the standard error. Data represent the results of four independent experiments (n = 2 or 3) under the same conditions.*

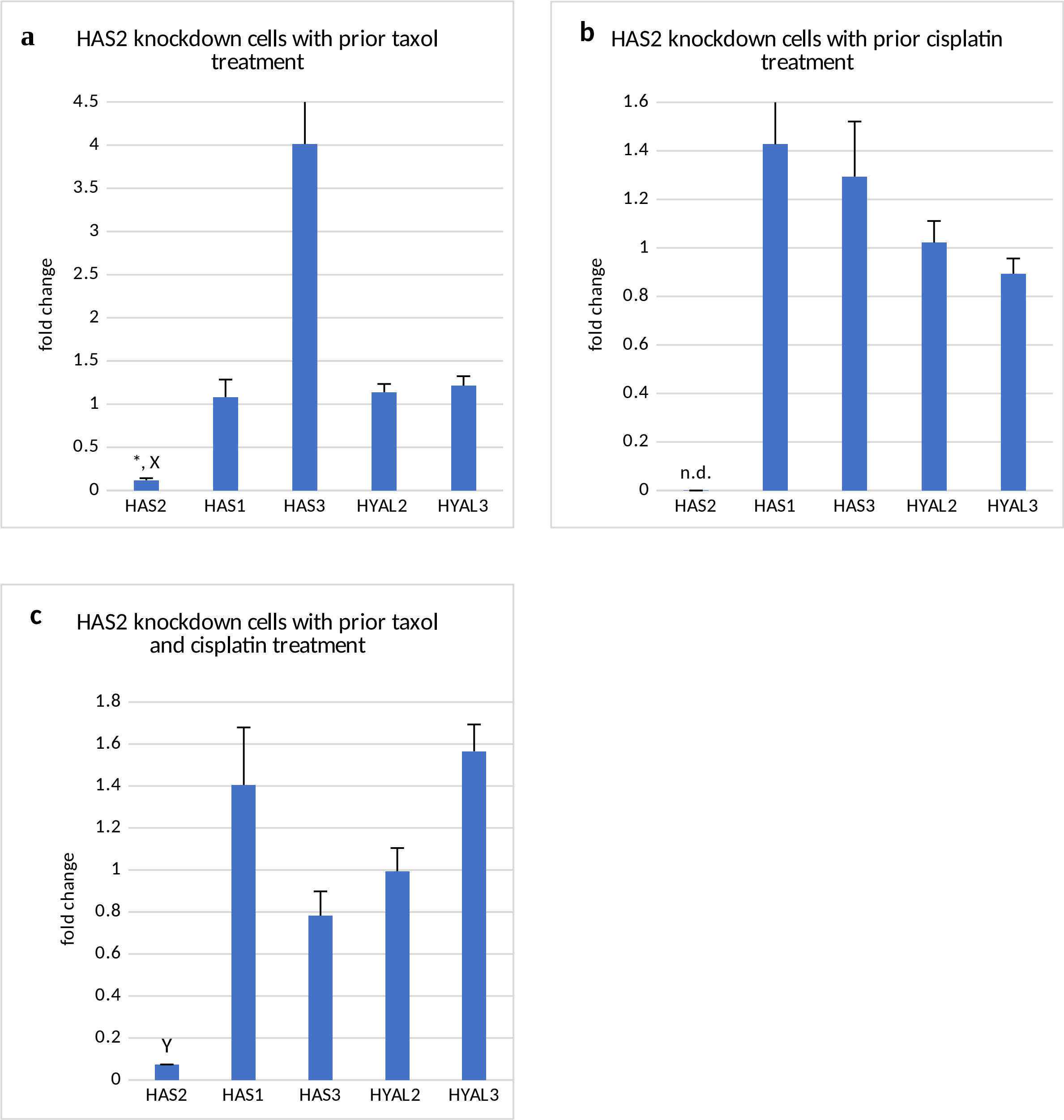

Supplementary Figure S4 *HAS2 knockdown in SKOV3 cells and its influence on the expression of HAS1, HAS3, HYAL2 and HYAL3 measured by qPCR. Cells were treated with different chemotherapy regimens 24 hours after transfection. The mean value was given with the standard error. Data represent the results of three independent experiments (n = 3) under same conditions. *p ≤ 0.05* ***a****: treatment with 8000 nM taxol, X = in 4 of 6 samples HAS2 expression was not detectable, mean value was built out of the other 2 values,* ***b****: treatment with 53.3244 nM cisplatin, n.d. = in 5 of 6 samples expression of HAS2 was not detectable, one value was measured with a fold of 0.72, this value was excluded.* ***c****: treatment with the combination of 42,86 nM taxol and 38.08 nM cisplatin, Y = in 5 of 6 samples expression of HAS2 was not detectable*

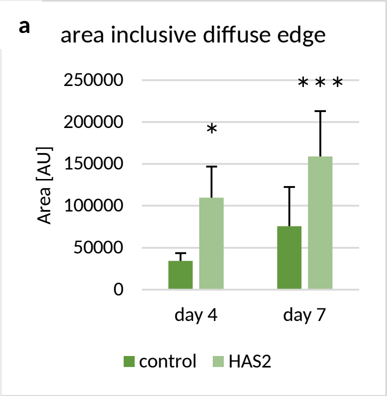

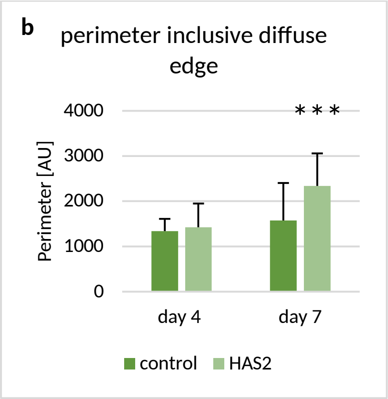

c

	diffuse edge	no diffuse edge
control day 4	25%	75%
control day 7	54%	46%
HAS2 day 4	76%	24%
HAS2 day 7	100%	0%

Supplementary Figure S5. *Hanging drop method was used to show differences in cell cohesion and sphere formation capability of HAS2 knockdown SKOV3 cells compared to control SKOV3 cells. The area or perimeter of spheres respectively the diffuse edge of HAS2 knockdown and control cells was measured at day 4 and day 7.* ***a,b****) area and perimeter of the spheres including the diffuse edge. Data represent mean ± SD from four different experiments which were done under the same conditions. *p ≤ 0.05, ***p ≤ 0.0001* ***c****) The frequency of spheres that built a diffuse edge is shown in %. The values were built out of data of 4 experiments á 12 drops for HAS2 knockdown and control cells (n=48). AU = arbitrary unit.*

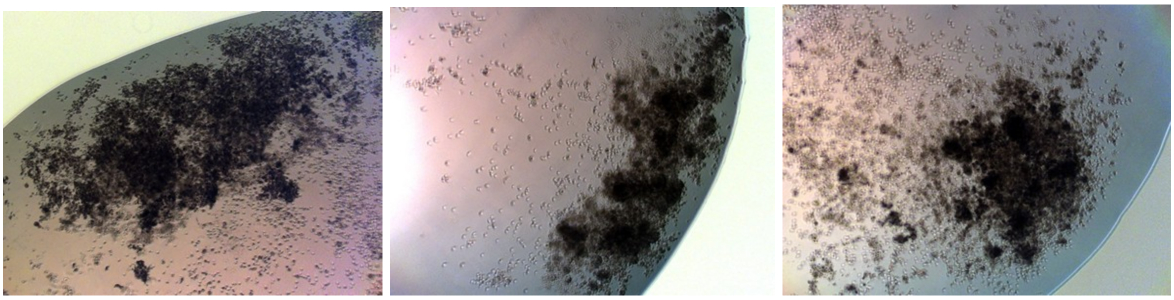

Supplementary Figure S6. *Representative micrographs of SW626 cells failing to form proper spheroids in the hanging drop assay.*

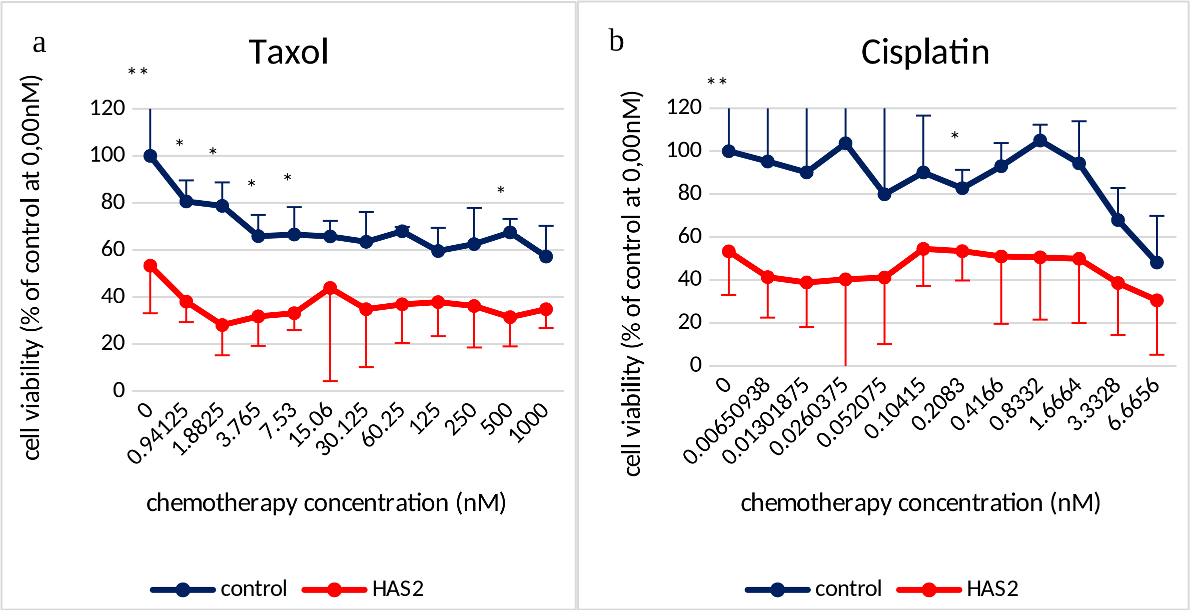

Supplementary Figure S7. *Viability of SKOV3 HAS2 knockdown cells and control cells at different chemotherapy treatments, measured by MTT assay. All values are given in % based on the concentration of control cells at 0,00 nM chemotherapy treatment. Results represent mean value ± SD for three experiments under the same conditions. *p ≤ 0.05, **p ≤ 0.001.* ***a****: treatment with taxol,* ***b****: treatment with cisplatin*

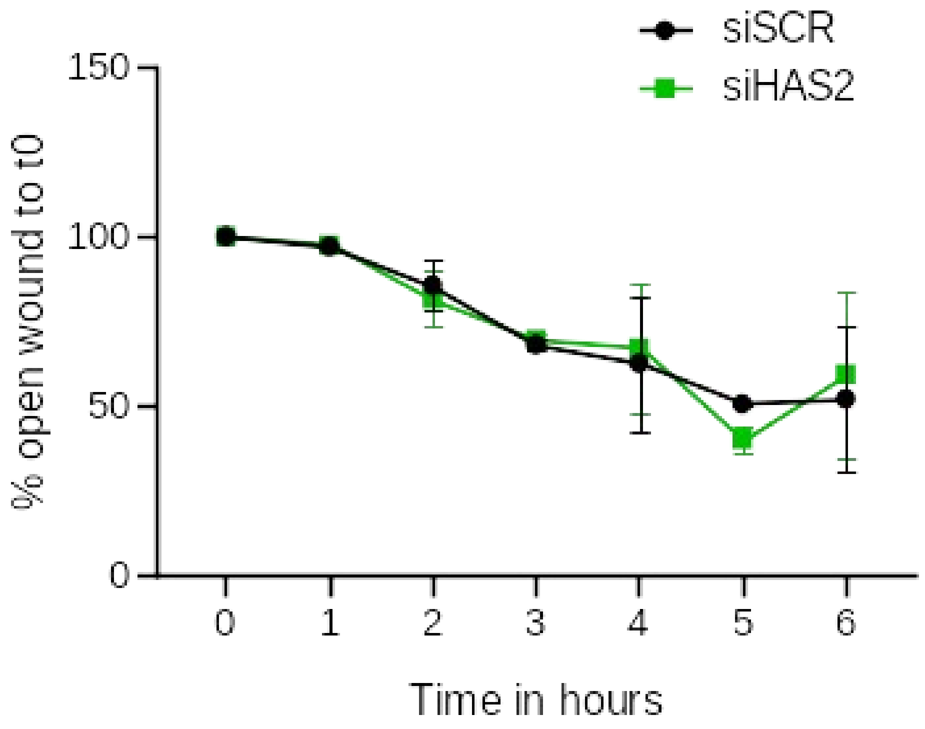

Supplementary Figure S8. *Cell migration of SKOV3 HAS2 knockdown cell s(siHAS2) and control cells (siSCR) at 1 hour intervals, measured by wound healing assay. Results are presented as a percentage of wound closure and represent mean value ± SD for three experiments under same conditions. No significant differences are seen between treatment groups.*

Table S1: *Sequences of primer pairs*

SYBR-Green

Actin	Forward: TCA AGA TCA TTG CTC CTC CTG AG<br>Reverse: ACA TCT GCT GGA AGG TGG ACA
HAS1	Forward: CTG CGA TAC TGG GTA GCC TTC A<br>Reverse: CCA GGA ACT TCT GGT TGT ACC AG
HAS3	Forward: ACT CTG CAT CGC TGC CTA CC<br>Reverse: TAC ATG ACC TCA CGC TTG CC
HYAL2	Forward: GGA CCT CAT CTC TAC CAT TGG C<br>Reverse: CTT TGA GGT ACT GGC AGG TCT C
HYAL3	Forward: GCA GTC CAT TGG TGT GAG TGC A<br>Reverse: CCA AGG TGT CCA CCA GGT AGT C
CD44	Forward: GCC CAT TGT TCA TTC TTG TGC T<br>Reverse: AAA CCA GAG GAA GGG TGT GCT C
RHAMM	Forward: GGC TGG GAA AAA TGC AGA GGA TG<br>Reverse: CCT TTA GTG CTG ACT TGG TCT GC
VCAN	Forward: GTG CCT TTC CTA TCA CCT CGA GAA<br>Reverse: AAA TGA CTG AAC GGT GGT CCA A

Taq-man assay code numbers (Thermo Fisher)

Actin	hs 99999903 m1
HAS2	hs 00193435 m1

Table S2: *Correlation between the expression of hyaluronan synthases and hyaluronidases and the overall survival (OS) or progression free survival (PFS) of ovarian cancer patients. Subgroup analysis was done referred to the chemotherapy with taxol or cisplatin. Data were analyzed by the Kaplan-Meier-Plotter. Number of cases, HR and p-value are given. Statistically significant values are marked in bold typing.*

		**OS**			**PFS**		
**genes**	**chemotherapy**	**cases**	**HR**	**p-value**	**cases**	**HR**	**p-value**
**HAS 1**	platin	1409	1.06	0.41	1259	1	0.96
	taxol	793	1.12	0.25	715	1.26	**0.007**
**HAS 2**	platin	1409	1.27	**0.0007**	1259	1.05	0.46
	taxol	793	1.22	**0.041**	715	1.23	**0.016**
**HAS 3**	platin	478	0.96	0.76	502	1.11	0.29
	taxol	357	0.88	0.38	381	0.99	0.94
**HYAL1**	platin	1409	1.08	0.31	1259	0.95	0.44
	taxol	793	1.16	0.12	715	1	0.97
**HYAL2**	platin	1409	0.98	0.79	1259	0.88	0.063
	taxol	793	0.92	0.39	715	0.93	0.41
**HYAL3**	platin	1409	0.85	**0.02**	1259	0.8	**0.0006**
	taxol	793	0.84	0.064	715	0.9	0.23
**HYAL4**	platin	1409	0.94	0.42	1259	0.9	0.12
	taxol	793	0.88	0.19	715	0.96	0.66
**PH20**	platin	1409	1.02	0.8	1259	0.94	0.31
	taxol	793	1.15	0.14	715	1.08	0.4
**HYALP1**	platin	478	0.89	0.34	502	0.89	0.24
	taxol	357	1	0.98	381	0.92	0.48

Table S3: *Correlation between the expression of HYALP1 and the overall survival (OS) or progression free survival (PFS) of ovarian cancer patients. Distinction was made between the subgroup's histology (serous or endometrioid), staging (I + II or III + IV), grading (I+ II or III) and chemotherapy (taxol + platin). Data were analyzed by the Kaplan-Meier-Plotter. Number of cases, HR and p-value are given. Statistically significant values are marked in bold typing.*

			**OS**			**PFS**		
**genes**			**cases**	**HR**	**p-value**	**cases**	**HR**	**p-value**
**HYALP1**	**histology**	serous	523	0.94	0.56	483	0.94	0.55
		endometrioid	30	1.4	0.74	44	0.73	0.55
	**staging**	I + II	83	1.07	0.9	115	1.26	0.53
		III + IV	487	0.89	0.3	494	0.9	0.31
	**grading**	I + II	203	0.59	**0.011**	189	0.94	0.74
		III	392	0.93	0.57	315	0.91	0.44
	**chemotherapy**	taxol + platin	356	1	1	380	0.92	0.45
